# Enzymatic Hydrolysis of Human Milk Oligosaccharides. The Molecular Mechanism of *Bifidobacterium Bifidum* Lacto-*N*-biosidase

**DOI:** 10.1021/acscatal.2c00309

**Published:** 2022-04-06

**Authors:** Irene Cuxart, Joan Coines, Oriol Esquivias, Magda Faijes, Antoni Planas, Xevi Biarnés, Carme Rovira

**Affiliations:** † Departament de Química Inorgànica i Orgànica & IQTCUB, Universitat de Barcelona, Martí i Franquès 1, 08028 Barcelona, Spain; ‡ Laboratory of Biochemistry, Institut Químic de Sarrià, Universitat Ramon Llull, Via Augusta 390, 08017 Barcelona, Spain; § Institució Catalana de Recerca i Estudis Avançats (ICREA), Passeig Lluís Companys, 23, 08020 Barcelona, Spain

**Keywords:** human milk oligosaccharides, lacto-*N*-biosidase, carbohydrates, glycosidases, quantum mechanics/molecular mechanics, metadynamics

## Abstract

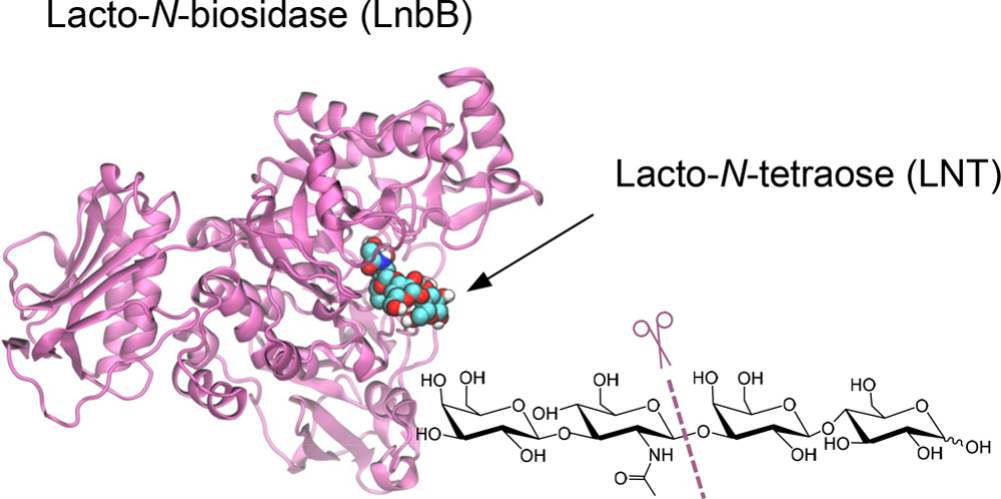

*Bifidobacterium bifidum* lacto-*N*-biosidase (LnbB) is a critical enzyme for the degradation of human milk oligosaccharides in the gut microbiota of breast-fed infants. Guided by recent crystal structures, we unveil its molecular mechanism of catalysis using QM/MM metadynamics. We show that the oligosaccharide substrate follows ^1^*S*_3_/^1,4^*B* → [^4^*E*]^‡^ → ^4^*C*_1_/^4^*H*_5_ and ^4^*C*_1_/^4^*H*_5_ → [^4^*E*/^4^*H*_5_]^‡^ → ^1,4^*B* conformational itineraries for the two successive reaction steps, with reaction free energy barriers in agreement with experiments. The simulations also identify a critical histidine (His263) that switches between two orientations to modulate the p*K*_a_ of the acid/base residue, facilitating catalysis. The reaction intermediate of LnbB is best depicted as an oxazolinium ion, with a minor population of neutral oxazoline. The present study sheds light on the processing of oligosaccharides of the early life microbiota and will be useful for the engineering of LnbB and similar glycosidases for biocatalysis.

Human milk oligosacharides (HMOs) comprise a group of structurally complex, unconjugated glycans that are highly abundant in human milk. They play a crucial role in defining the intestinal microbioma of infants,^1−3^ conferring them a protective barrier against infections, immunomodulation effects, and nutritive support that infants with poor access to breast milk do not acquire in the first years of life.^4^ Hence, there is great interest in the enzymes responsible of the synthesis, degradation, and modification of HMOs for nutrition-related applications, such as supplements for infant formula milks.^5^

Lacto-*N*-biosidase from *Bifidobacterium bifidum* (LnbB) is a critical enzyme for the degradation of HMOs in the gut microbiota of breast-fed infants.^6^ First identified in 2008,^6^ LnbB hydrolyzes HMOs from their nonreducing end, releasing lacto-*N*-biose (LNB), a disaccharide of galactose and *N*-acetylglucosamine (Gal-β-1,3-GlcNAc) (Figure 1a) that is the main core of the most predominant HMOs.^7^

**Figure 1:**
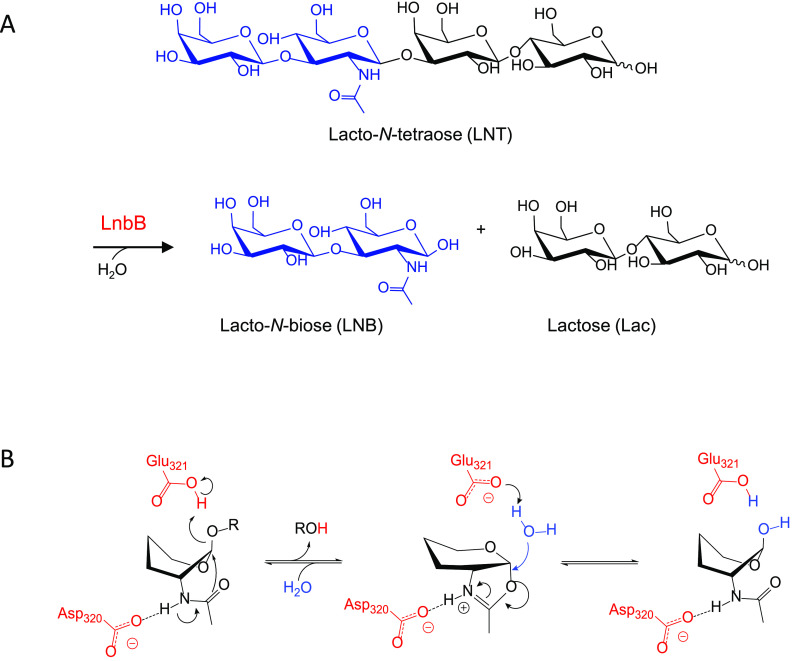
(A) Hydrolysis of the human milk oligosaccharise lacto-*N*-tetraose (LNT) catalyzed by *Bifidobacterium bifidum* lacto-*N*-biosidase (LnbB). (B) Substrate-assisted reaction catalyzed by LnbB.

LnbB is a member of family 20 retaining glycoside hydrolases (GH20).^8^ Enzymes of this family present diverse domain organizations that may include catalytic and noncatalytic domains.^9^ LnbB in particular contains a lectin domain that extends toward the catalytic site (Figure S1). The first structures of LnbB complexes were obtained by Fushinobu and co-workers in 2013.^10^ The structure of LnbB in complex with LNB (i.e., a product complex) and, in particular, that of the complex with a LNB-thiazoline inhibitor, were consistent with substrate-assisted catalysis (Figure 1b),^11^ also named as *neighboring group participation*. In this mechanism, the saccharide at the *–1* subsite (hereafter named as the “reactive sugar”) makes use of its acetamido (NHAc) substituent at C2 to perform nucleophile attack on the anomeric carbon, while an acid/base residue protonates the leaving group. The reaction is assisted by an aspartate residue that interacts with the acetamido NH group, promoting the formation of an oxazoline or an oxazolinium ion, depending on the location of the acetamido proton, which is not known, during catalysis. Mutagenesis studies of LnbB have demonstrated that both the acid/base and the assisting residue (Glu321 and Asp320, respectively) are required for catalysis (the Glu321Ala variant is inactive enzyme, while Glu320Ala retains 1% of the activity of the wild-type enzyme).^10,12^ Substrate-assisted catalysis has been also described for other GHs such as GH18 chitinases,^13^ GH20 hexosaminidases,^11^ GH84 *O*-GlcNAcases,^14,15^ GH56 hyaluronidases,^16^ GH85 endo-β-*N*-glucosaminidases,^17^ and GH123 endo-β-*N*-galactosaminidases.^18^

On the basis of the structures of LnbB complexes, and by analogy with other GH20 enzymes,^19−22^ Fushinobu and co-workers hypothesized a substrate conformational itinerary in which both transition states of the chemical reaction (Figure 1b) feature a ^4^*E* conformation of the reactive sugar. In particular ^1,4^*B* → [^4^*E*]^‡^ → ^4^*C*_1_ and ^4^*C*_1_ → [^4^*E*]^‡^ → ^4^*E* itineraries were proposed for the first and second chemical steps, respectively. As previously discussed,^10^ LnbB differs from other GH20 family members such as hexosaminidases in that it does not cleave oligosaccharides at the terminal end but it releases a disaccharide (Figure 1a). As such, LnbB is the only GH20 enzyme that has evolved an enzyme subsite (*−2* subsite) to accommodate an additional monosaccharide (Figures 2 and S1). Therefore, the details of the reaction mechanism of LnbB, specifically the conformational itinerary of the reactive sugar during catalysis and the protonation state of the reaction intermediate could also differ from other GH20 enzymes and even from other GHs following substrate-assisted catalysis.

**Figure 2:**
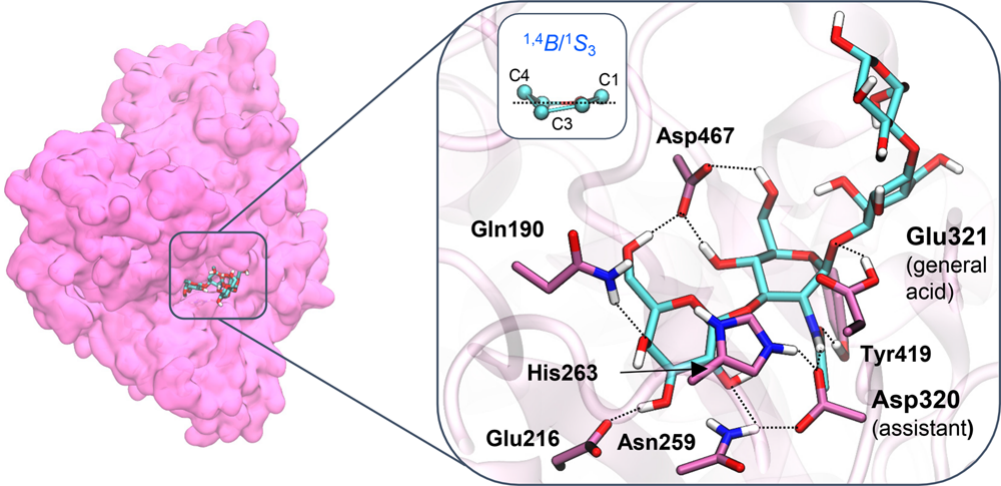
Structure of LnbB in complex with lacto-*N*-tetraose (LNT) obtained from MD and QM/MM MD simulations.

Several theoretical studies of substrate-assisted mechanisms in GHs using quantum mechanics/molecular mechanics (QM/MM) methods have been reported. These include studies of the reaction mechanism of family 18 chitinases A and B,^23−25^ an investigation of the second step of the reaction catalyzed by family 84 *O*-GlcNAcase,^26^ and the characterization of the reaction intermediate of family 20 hexosaminidase.^27^ These studies confirmed the substrate-assisted mechanism for these enzymes, and their results suggest that the protonation state of the reaction intermediate (oxazoline or oxazolinium ion-type) depends on the specific architecture of the active site, which differs in each case. To the best of our knowledge, the molecular mechanism of catalysis by LnbB or any enzyme hydrolyzing human milk oligosaccharides has not been unveiled by theoretical methods yet.

Guided by recent crystallographic structures of LnbB complexes, here we investigate the catalytic reaction mechanism of LnbB using QM/MM metadynamics. We provide a detailed atomistic view of the reaction coordinate, including the conformational catalytic itinerary of the substrate and the protonation dynamics of the reaction intermediate. Our simulations also reveal the critical role of an active site histidine that switches between two conformations, interacting with either of the two catalytic residues during the chemical reaction. This serves to modulate the p*K*_a_ of the acid/base residue, facilitating catalysis.

A Michaelis complex structure of LnbB with its natural substrate, which could be used to build an initial model for the simulations, is not available. However, there is a structure of LnbB in complex with a Gal-β-1,3-GlcNAc-thiazoline inhibitor that mimics the substrate of the reaction at the reaction intermediate.^10^ We used this structure to reconstruct the Michaelis complex of LnbB with a lacto-*N*-tetraose (LNT) substrate using docking and molecular dynamics (MD) simulations (see details in the SI). Remarkably, the reactive sugar, initially in a ^4^C_1_ conformation, spontaneously evolved toward a conformation between ^1,4^B and ^1^S_3_ during the classical MD simulations (Figures S2–S4). Such a conformation keeps the scissile glycosidic bond of the reactive sugar in an axial orientation, which is a prerequisite for efficient catalysis in GHs.^28−30^

The structure obtained from classical MD simulations was refined by QM/MM MD. The CPMD code,^31,32^ which combines Car–Parrinello MD,^33^ based on Density Functional Theory (QM atoms), with the AMBER energy function (MM atoms), was used. The QM region was taken as to include the saccharide units at the *–2*, *–1*, and part of the *+1* subsites, plus the catalytic residues Glu321 and Asp320 (67 QM atoms, 111345 MM atoms, Figure S5). Such a methodology has been previously adopted with success to model catalysis in carbohydrate-active enzymes,^34−41^ including those that operate via a substrate-assisted mechanism.^23,26^

A close view of the active site of the enzyme–substrate complex obtained by QM/MM MD is shown in Figure 2. The acid/base residue (Glu321) turned out to be quite mobile, with two orientations sampled along the simulation, as was also observed in the classical MD simulation (Figure S6). This is a consequence of the weak interaction between the solvent-exposed carboxylic acid group of the acid/base residue with the substrate. A similar scenario, with the acid/base residue adopting more than one conformation, was previously observed in other GHs.^34,41^ In contrast, the assisting residue (Asp320) is quite fixed in place, forming a strong interaction with the NH of the substrate acetamido group (Figure 2).

To model the first step of the enzymatic reaction, i.e., the formation of the oxazoline or oxazolinium ion intermediate, we used QM/MM metadynamics with two collective variables (CVs) (Figure 3a). The first CV was taken to include the distance from the anomeric carbon to the NHAc carbonyl oxygen and the glycosidic bond distance (CV1 = *d*_C1–O_NAc__ – *d*_C1–O1_). This CV accounts for the nucleophilic attack by the NHAc carbonyl oxygen atom on the anomeric carbon and leaving group departure. The second CV was selected as involving the O–H distance of the acid/base carboxylic acid and its hydrogen bond distance with the glycosidic oxygen (CV2 = *d*_(O–H)_Glu321__ – *d*_O1–H_Glu321__). Thus, the second CV quantifies the degree of protonation of the glycosidic oxygen. The reaction free energy surface (FES) obtained from the simulation (Figure 3a) shows three energy minima, corresponding to the Michaelis complex (MC and MC′), and the reaction intermediate (INT). The two minima in the Michaelis complex region (MC and MC′ in Figure 3a) differ in the orientation of the acid/base residue, which can interact either with the glycosidic oxygen (MC) or with the 2-OH substituent of the galactose saccharide at the *+1* subsite (MC’) (Figure 3b). This is consistent with the dynamical behavior of this residue observed in the QM/MM MD simulations (Figure S6). The FES exhibits only one transition state (Figure 3a), which is indicative of a concerted S_N_2 reaction. The reaction free energy barrier (15.3 kcal/mol) agrees with the value that can be estimated from the experimental reaction rate (*k*_cat_ = 42.1 ± 0.8 s^–1^; Δ*G*^‡^ ≈ 15 kcal/mol).^10^

**Figure 3:**
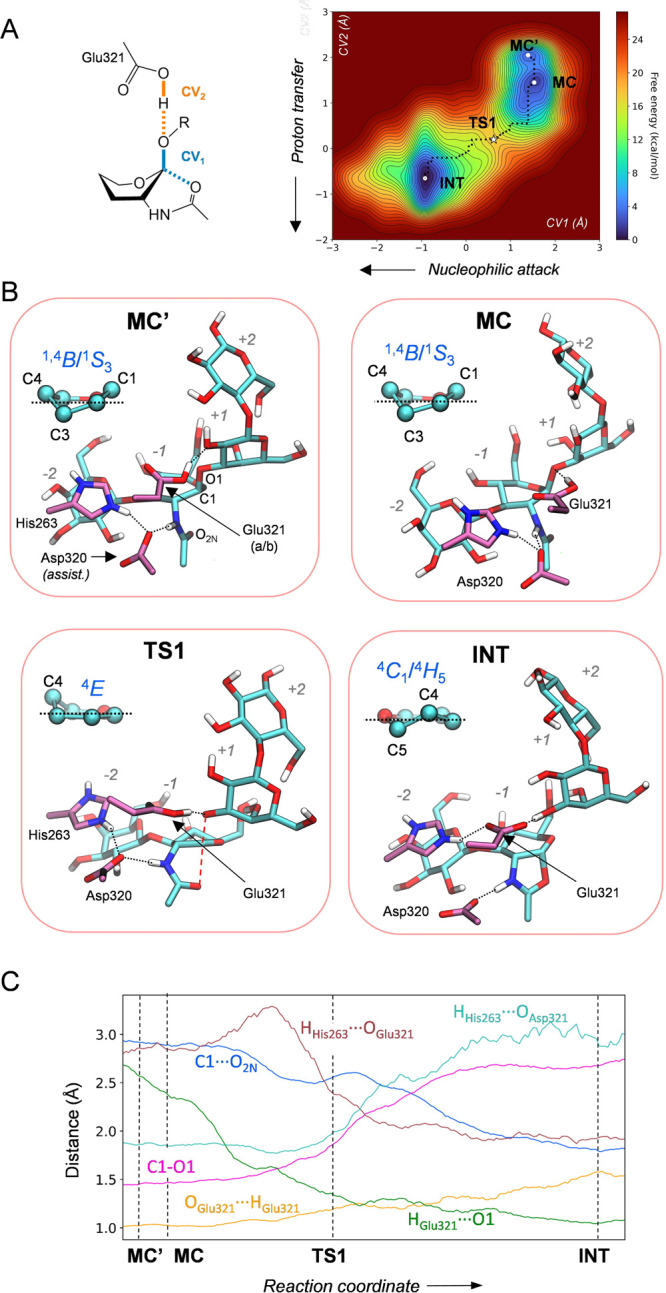
First step of the chemical reaction catalyzed by LnbB. (A) Reaction free energy surface as a function of depicted CVs. (B) Most relevant states along the reaction coordinate. (C) Evolution of relevant distances along the reaction coordinate.

Analysis of the enzyme structure along the minimum free energy pathway provides an atomic picture of the active site dynamics along the reaction coordinate (Figure 3). The reaction starts by elongation of the glycosidic bond, while the acetamido oxygen atom (O) approaches the anomeric carbon (C1). Simultaneously, the acid/base residue (Glu321) transfers a proton (H) to the glycosidic oxygen (O1). The transition state (TS1) features a planar configuration around the anomeric carbon, with the reactive sugar adopting a ^4^E conformation, as predicted by Fushinobu and co-workers on the basis of crystal structures.^10^ The glycosidic bond is significantly stretched (C1–O1 = 1.99 ± 0.15 Å; Table S1 and Figure 3c), while the acetamido oxygen is not yet attacking the anomeric carbon (C1···O_2N_ = 2.59 ± 0.13 Å) at TS1, which thus exhibits dissociative character. The proton of the acid/base residue is partially transferred at the TS (O1···H = 1.39 ± 0.05 Å; H–O_Glu321_ = 1.14 ± 0.04 Å), indicating that proton transfer happens together with glycosidic bond cleavage. This is in contrast with substrates with nonsaccharide aglycon groups such as methylumbelliferyl or *p*-nitrophenyl (pNP),^34,41^ for which proton transfer occurs after the TS or even once the reaction intermediate forms. In these cases, the lower p*K*_a_ of the aglycon with respect to a saccharide leaving group facilitates the cleavage of the glycosidic bond. In contrast, a sugar leaving group (p*K*_a_ ≈ 12) requires strong proton assistance form the acid/base residue. Overall, the first step of the reaction catalyzed by LnbB can be described as a proton-assisted S_N_2 and D_N_A_N_ type,^42^ with the reactive sugar delineating a ^1^*S*_3_/^1,4^*B* → [^4^*E*]^‡^ → ^4^*C*_1_/^4^*H*_5_ itinerary.

The LNB fragment of the LNT substrate remains tightly bound in the active site during the reaction, via enzyme–substrate interactions with the residues at the *–1* and *–2* subsites, whereas the lactose at the reducing end remains solvent-exposed (Figure 3b). Three negatively charged residues (Asp467, Glu216, and Asp320), as well as two neutral residues (Asn259 and Tyr419) (Figure 2) are engaged in interactions with hydroxyl groups and the NHAc group of the LNB all along the reaction coordinate. In contrast, there is one active site residue that does not keep the same interaction pattern along the reaction coordinate. Specifically, His263 moves to change the hydrogen bond partner from the assisting residue to the acid/base residue as the reaction progresses. In the reactants complex, His263 forms a hydrogen bond with the assisting residue (Asp320), which in turn interacts with the substrate NHAc (Figure 3b, MC and MC′ states). This interaction lowers the p*K*_a_ of Asp320, keeping the H atom of the substrate acetamido group attached to the LNT substrate. However, once the glycosidic bond stretches and the acid/base residue acquires a negative charge (by partial transfer of the proton to the glycosidic oxygen, O1), His263 departs from Asp320 (Figure 3b, INT state) and moves toward the acid/base residue (Glu321), stabilizing its negative charge and thus preparing the active site for the second step of the enzymatic reaction. The hydrogen bond switch of His263 makes the NHAc proton more prone to be transferred to Asp320 (the N–H distance increases by 0.1 Å from MC to INT, Table S1).

It is also interesting to analyze the protonation state of the reaction intermediate, which has been described as either an oxazolinium ion or a neutral oxazoline in GHs following substrate-assisted catalysis. Previous QM/MM studies showed that the intermediate of GH18 Chitinase B features a neutral oxazoline,^23^ while a positively charged oxazolinium ion was found for GH84 *O*-GlcNAcase.^26^ In the case of LnbB, the dynamic motion of His263, moving away from Asp320 upon formation of the reaction intermediate, is expected to rise the p*K*_a_ of Asp320 and increase the population of the oxazoline form. In fact, our QM/MM MD simulations show that, even though the NHAc proton remains most of the time bound to the N atom, it undergoes transitions toward Asp320. Consistently, a metadynamics simulation of the proton transfer coordinate (Figure S8) shows that both states (oxazolinium ion and oxazoline) differ by less than 1 kcal/mol, with the oxazolinium ion form being favored. Therefore,^28^ the reaction intermediate of LnbB is best described as an oxazolinium ion, with a minor population (≈30%) of neutral oxazoline.

The structure of the reaction intermediate obtained from the simulations is in good agreement with the crystal structure of LnbB in complex with LNB-thiazoline (PDB 4JAW)^10^ (Figure S9). This structure, which represents a state in which the lactose aglycon has already exited the active site, was used as the starting point to model the second step of the enzymatic reaction. The ability of the enzyme to hydrolyze the C1–O_2N_ bond depends on the presence of a well oriented water molecule in the active site. Analysis of the water dynamics during a classical MD simulation shows that indeed water molecules go in and out of the active site, with one water molecule frequently siting close to the anomeric carbon of the GlcN-oxazolinium ion (C1···O_w_ < 4 Å) (Figure 4). Most importantly, such a water molecule sometimes interacts with the acid/base residue in a suitable orientation for nucleophilic attack on the C1 atom, with one of the lone pairs pointing toward the C1 atom (Figure 4). We took one of such configurations to initiate the simulations of the second step of the enzymatic reaction. The hydrolysis reaction was modeled with QM/MM metadynamics using two collective variables, accounting for the nucleophilic attack of the water molecule onto the anomeric carbon (CV1 = *d*_C1–O_*w*__ – *d*_C1–O_), and the proton transfer between the nucleophilic water and the acid/base residue (CV2 = *d*_O_Glu321_*–*H_*w*__ – *d*_H_w_–O_*w*__). The FES obtained from the simulation is consistent with a concerted and dissociative S_N_2 reaction (the C1–O_2N_ bond breaks before the C1–O_w_ bond starts to form) in which the reactive sugar follows a ^4^*C*_1_/^4^*H*_5_ → [^4^*E*]^‡^ → ^1,4^*B* conformational itinerary (Figure 5). The computed reaction free energy barrier (11.7 kcal/mol) is lower than the energy barrier for the first step by 3.3 kcal/mol, indicating that the hydrolysis step is not rate-limiting. Interestingly, the motion of His263 mirrors that of the first step. At the reaction intermediate, His263 prefers to interact with the negatively charged acid/base residue (Glu321) (Figure 5b, INT state). However, as the water molecule starts attacking the anomeric carbon and delivers a proton toward Glu321, which then becomes a neutral residue, His263 flips back to interact with the assisting residue Asp320. As a result, the active site recovers the original configuration of the Michaelis complex and the enzyme is ready for another round of catalysis (Figure 5b, P state). Therefore, His263 exerts its modulatory role during the complete reaction coordinate, switching its interaction between the two catalytic residues according to its protonation state. Interestingly, mutation of His263 to nonpolar residues such as Phe or Ala has been found to decrease significantly the catalytic rate of LnbB with LNB-pNP substrates (by 2 orders of magnitude), indicating a crucial role in catalysis.^10,12^ Our simulations reveal the molecular basis of this experimental observation, showing that His263 assists catalysis by changing the hydrogen bond partner between Asp320 and Glu321 as the reaction progresses.

**Figure 4:**
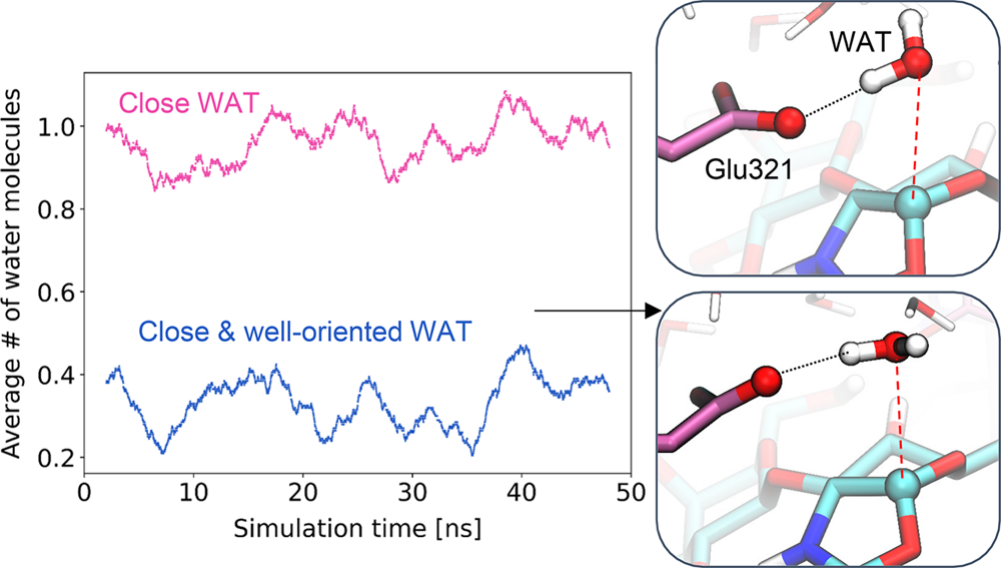
Analysis of the dynamics of water molecules in the active site at the reaction intermediate. Multiple replicas of the simulation (total 300 ns) gave similar results.

**Figure 5:**
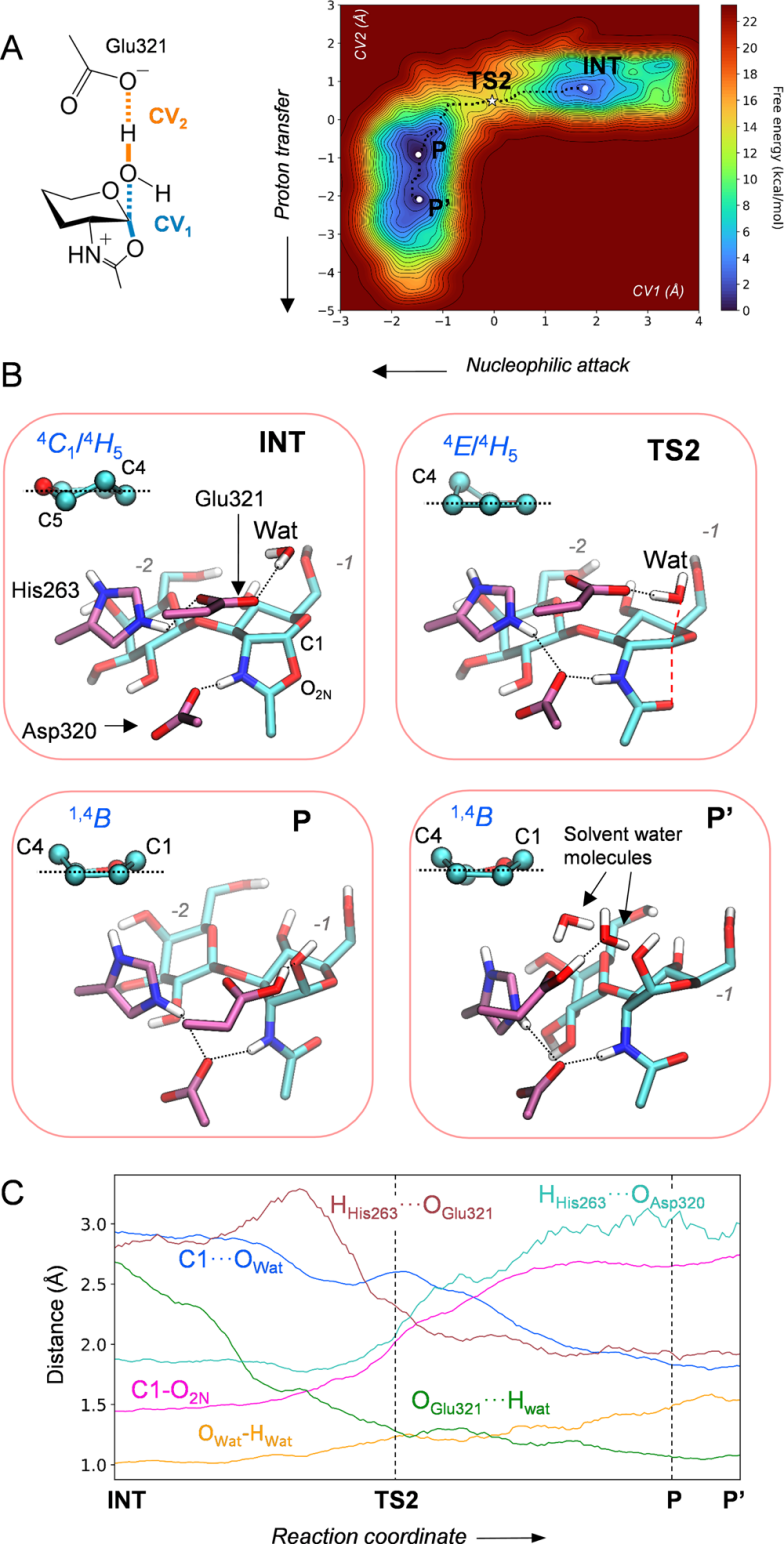
Second step of the chemical reaction catalyzed by LnbB. (A) Collective variables used to drive the metadynamics bias and reconstructed reaction free energy surface. (B) Main states along the reaction coordinate. (C) Evolution of relevant distances along the reaction coordinate.

The catalytic role of His263 that emerges from the simulations deserves further attention. Most classical retaining GHs have a residue in the active site that modulates the p*K*_a_ of the acid/base residue, lowering it upon formation of the covalent glycosyl–enzyme intermediate.^43,44^ The identity of the p*K*_a_ modulator in retaining GHs following a substrate-assisted mechanism is less clear-cut. It was found in GH18 chitinases that the assisting residue itself (Asp142, equivalent to Asp320 in LnbB) plays the role of such p*K*_a_ modulator, as the two catalytic residues (Asp142 and Glu 144) are engaged in a hydrogen bond that connects the NHAc group with the glycosidic bond. This is possible in GH18 because both catalytic residues are not consecutive in the sequence. In LnbB, however, both catalytic residues are consecutive (Asp320 and Glu321) and thus, by construction, can hardly form a hydrogen bond. In this case, an additional residue modulating the p*K*_a_ of the catalytic residues is needed, as in classical retaining GHs. Our simulations clearly show that His263 plays this role in LnbB and this can possibly be extended to GH20 enzymes in general, all of them exhibiting a conserved histidine in the active site.^10^

In summary, by means of MD and QM/MM metadynamics methods we have unraveled the mechanism of action of LnbB, one of the crucial enzymes for the hydrolysis of HMOs. We reveal that substrate-assisted catalysis is facilitated by the motion of an active site histidine that dynamically modulates the p*K*_a_ of the acid/base catalytic residue. In addition, we provide the substrate conformational itinerary and the structure of the transition states along the reaction coordinate, which will be useful for the engineering of LnbB and related β-hexosaminidases for biocatalytic applications. These results increase our understanding of GHs following substrate-assisted mechanisms and can aid the engineering of the enzyme for the synthesis of HMOs for infant formula milk.^12,45,46^

## Supporting Information Available

Computational modeling of the reaction steps, tables of active site distances, figures of structures, RMSD evolution of the backbone C atoms along, conformations of the pyranose sugar, evolution of the collective variables, and free energy profile, and Cartesian coordinates (PDF)
