# Supplementary material for: Enzymatic Hydrolysis of Human Milk Oligosaccharides. The Molecular Mechanism of Bifidobacterium Bifidum Lacto-N-biosidase
Source: ACS Catal. 2022 Apr 6;12(8):4737–43. doi: 10.1021/acscatal.2c00309 (PMC9016705; doi:10.1021/acscatal.2c00309)
Supplement: Supplementary file 1 — cs2c00309_si_001.pdf [file cs2c00309_si_001.pdf]

SUPPORTING INFORMATION

Enzymatic Hydrolysis of Human Milk Oligosaccharides. The Molecular Mechanism of *Bifidobacterium Bifidum* Lacto-*N*-Biosidase

Irene Cuxart[1], Joan Coines[1,4], Oriol Esquivias[1], Magda Faijes[2], Antoni Planas[2], Xevi Biarnés[2], Carme Rovira[1,3,*]

[1]Departament de Química Inorgànica i Orgànica & IQTCUB, Universitat de Barcelona, Martí i Franquès 1, 08028 Barcelona, Spain. [2]Laboratory of Biochemistry, Institut Químic de Sarrià, Universitat Ramon Llull, Via Augusta 390, 08017 Barcelona, Spain.[3]Institució Catalana de Recerca i Estudis Avançats (ICREA), Passeig Lluís Companys, 23, 08020 Barcelona, Spain. [4]Present address: NBD Nostrum Biodiscovery, Parc Científic de Barcelona, Baldiri Reixac 10, 08028 Barcelona, Spain.

Corresponding Author

[*]Carme Rovira (c.rovira@ub.edu)

Contents

1. Modeling of the first reaction step ………………………………………………….. S2

    1.1. System preparation ………………………………………………………… S2

    1.2. Classical molecular dynamics ……………………………………………... S2

    1.3. QM/MM molecular dynamics ……………………………………………... S3

    1.4. QM/MM metadynamics …………………………………………………… S3

2. Modeling of the second reaction step ……………………………………………... S4

    2.1. System preparation ………………………………………………………… S4

    2.2. Classical molecular dynamics ……………………………………………… S5

    2.3. QM/MM molecular dynamics ……………………………………………… S5

    2.4. QM/MM metadynamics ……………………………………………………. S6

3. References ……………………………………………………………………………. S7

4. Supporting tables ………...…………………………………………………….……... S11

5. Supporting figures ………...………………………………………………………… S13

6. Cartesian coordinates of the main states along the reaction pathway …………………S20

1. Modeling of the first reaction step

1.1. System preparation

The initial coordinates for the simulations were taken from the crystal structure of *Bifidobacterium bifidum*'s LnbB in complex with LNB-thiazoline (PDB accession code 4JAW, resolution 1.80 Å)[1] (Figure 1). The model was built using chain A. Residues 30 to 40, which they were added in the cloning process for a better packing of the construct, were removed, as well as the LNB-thiazoline ligand. The protonation states of aspartate, glutamate and histidine residues was assigned considering the environment of each residue and the optimal pH for the enzyme activity (pH 4.5) which was also tested with PropKa3 and H++ [2–4]. The assisting residue (Asp320) was considered as deprotonated, and the acid/base residue (Glu321) was taken in its protonated form. The LNT substrate was docked in the active site using the program AutoDock VINA[5](Figure S2). Different poses were generated and the best ones in terms of contacts with the residues of the -2 and -1 subsites (reproducing the ones observed in the X-ray structure of the enzyme with LNB-thiazoline, PDB 4JAW) were kept and submitted to classical MD simulations.

1.2. Classical molecular dynamics

The AMBER18[6] package was used in all classical molecular dynamics simulations. The system was solvated in a cubic cell of 33868 water molecules and 10 Cl^- anions were added to neutralize the system. The force fields FF99SB[7], GLYCAM_06j[8], and TIP3P [9] were used for the protein residues, the oligosaccharide substrate and the water solvent molecules, respectively.

Classical MD simulations started with energy minimization. First, only water molecules and $Cl^–$ anions were minimized (from residues 629 to 34507), followed by minimization of the whole system, using steepest descent and conjugate gradients methods. Next, the system was gradually heated to 300 K by increasing the temperature 100 K over 50 ps runs (only solvent and ions during the first 100 K, then the whole system until 100K, 200K and 300K), using a timestep of 1 fs. Once the system was at 300 K, the density was equilibrated in the NPT ensemble (2 ps). Finally, the simulation was extended in the NVT ensemble for 50 ns (Figure

S3) using a timestep of 2 fs. Restraints at interaction distances in the active site were used to stabilize the ligand at the binding site, which were gently released during the production run.

1.3. QM/MM molecular dynamics

QM/MM MD simulations were performed using the CPMD code, version 3.15.1 (http://www.cpmd.org). [10] A snapshot of the classical MD of the enzyme-substrate complex was used as starting point for QM/MM. The QM region was enclosed in a 15.09 × 19.84 × 18.52 Å cell with the side chains of the catalytic residues Asp320 and Glu321, the entire GlcNAc unit at the -1 subsite and part of the sugars at the *-2* and *+1* subsites, in total 67 atoms including 6 monovalent link atoms that were used to saturate the QM region[11] (Figure S5). The remaining 111345 atoms were treated with molecular mechanics (MM). A larger QM region including the positively charged His263 residue was also tested to make sure that there is no proton transfer between this residue and Asp320 in the Michaelis complex. QM/MM simulations were performed using the method developed by Laio and coworkers,[12] which combines Car-Parrinello MD with DFT.[13] The QM/MM interface was treated using the multilayer electrostatic coupling scheme developed by Laio and coworkers,[12] with NN, MIX and ESP radii of 9.52, 11.11 and 15.87 Å, respectively.[11] We used the Perdew-Burke-Ernzerhof (PBE) generalized gradient-corrected approximation for DFT[14], as it has been assessed a suitable option for describing carbohydrate conformations[15] and other glycoside hydrolases.[16–19] A 70 Ry kinetic energy cutoff we used for expanding Kohn-Sham orbitals in the plane-wave basis set, along with norm-conserving *ab initio* Trouiller-Martins pseudopotentials.[20] The fictitious electron mass was set at 600 au and the timestep at 0.12 fs. Geometry optimization was carried out with QM/MM with annealing of the ionic velocities until the nuclear gradient is lower than $5\cdot10^{-4}$ au. The Nosé Hoover thermostat[21] was used to heat the system to 300 K during a 5 ps MD run. The final snap-shot of the QM/MM MD simulation was taken to start metadynamics simulations.

1.4. QM/MM metadynamics

The first reaction step leading to the formation of the reaction intermediate was modeled by QM/MM metadynamics. The open-source, community-developed PLUMED library[22], version 2.3.3[23] was coupled to CPMD. Two collective variables that discriminate the broken and formed bonds during the reaction were used to build the bias and reconstruct the free

energy landscape. The first CV [($C1\cdots O_{2N}$) – ($C1\cdots O1$)] accounts for the intramolecular nucleophilic attack and glycosidic bond cleavage, whereas the second CV [($O1\cdots H_{Glu321}$) – ($H_{Glu321}\cdots O_{Glu321}$)] accounts for the protonation of the leaving group. The evolution of CVs along the simulation is shown in Figure S7. Gaussian-like biasing potential functions were added every 340 simulation steps (40.8 fs) with a height of 1 kcal/mol and a width of 0.2 Å (for both CVs). The simulation was stopped once recrossing over the transition state took place, as recommended for chemical reactions.[24] Trajectory analyses were performed with VMD version 1.9.3,[25] the CPPTRAJ[26] module of AMBER18 and PLUMED. The program MEPSAnd[27] was used to calculate the minimum free energy path over the free energy landscape.

A separate metadynamics simulation was performed to evaluate the energy barrier of the proton transfer between the NHAc group and the assisting residue (Asp320) at the reaction intermediate (INT). A single CV accounting for the proton transfer as the difference of the two proton transfer distances ($O_{Asp320}\cdots H$ and $H\cdots N$) was used. The deposition time, Gaussian height and width were set at 130 MD steps (15.6 fs), 0.1 kcal/mol and 0.1 Å, respectively. The simulation was stopped once the oxazoline and oxazolinium ion states were sampled several times. The reconstructed free energy profile is depicted in figure S8.

2. Modeling of the second reaction step

2.1. System preparation

The structure of the reaction intermediate was built from the PDB structure of LnbB in complex with LNB-thiazoline (Figure S1, PDB accession code 4JAW[1]), following the procedure described in section 1.1. The S atom at the five-member ring of LNB-thiazoline was substituted to O to generate LNB-oxazoline, which corresponds to the true reaction intermediate. Protonation states of titratable residues were assigned by inspecting the protein environment at pH 4.5, combined with pKa prediction programs PropKa3 and H^{++}. The acid/base residue (Glu321) was considered in its unprotonated form, consistent with its catalytic role. The pair LNB-oxazolinium ion^{+}/Asp320^{-} form was chosen as starting point for the reaction intermediate.

2.2. Classical molecular dynamics

The enzyme-intermediate complex was solvated in a cubic cell of 33883 water molecules and 9 Cl^- anions. AMBER 18 was used in all simulations, along with forcefields FF14SB[28] (protein) GLYCAM_06j (for the Gal part of LNB-oxazoline) and TIP3P (solvent). Parameters were assigned according to the GLYCAM_06j and GAFF[30] force fields, using the Antechamber suite[31] of AMBER18. RESP charges for the LNB-oxazolinium were obtained using Gaussian09[29] at the HF/6-31G* level of theory

Modeling of the reaction intermediate with classical MD was performed using the protocol described in section 1.2. A detailed analysis of the dynamics of the catalytic water molecules moving near the reactive center during the MD simulations was performed using an in-house Python3 program. To identify water molecules well-positioned for catalysis, a cutoff of 4 Å was chosen for the water nucleophilic attack distance at the C_1 and 2.2 Å for the hydrogen bond with the general base Glu321. One of the structures fulfilling these criteria was chosen as starting point for the QM/MM calculations.

2.3. QM/MM molecular dynamics

The QM region (56 atoms) was selected as including the side chains of Asp320, Glu321, the catalytic water, the GlcN-ox$^+$ sugar ring at the *-1* subsite and part of the Gal sugar at the *-2* subsite (Figure S5). The QM/MM boundary was treated with monovalent link atoms as previously described. The QM region was enclosed in a 16.50 x 19.97 x 15.39 Å cubic cell. The MM region contained 111354 atoms, which were treated classically. The protocol used for QM/MM MD simulations was the same as described above (section 1.3). The radii of the zones of electrostatic coupling was taken as 15.34 Å (ESP), 11.64 Å (MIX) and 8.46 Å (NN). The fictitious electron mass was set at 600 au and the timestep at 0.12 fs. A geometry optimization was carried out by annealing on the ionic velocities until the nuclear gradient is lower than $5 \cdot 10^{-4}$ au. The system was heated to 300 K by coupling it to a Nosé-Hoover thermostat for 4 ps.

2.4. QM/MM metadynamics

QM/MM metadynamics were used to model the second reaction step leading to product formation. CVs including broken and formed bonds were used. One CV was taken as

including the nucleophilic attack of water and the opening of the oxazolinium ring [CV1: =(O_w···C1) – (C1···O_{2N})]. The second CV was taken as including the deprotonation of the catalytic water molecule [CV2: (O_{Glu321} ··· H_w) – (H_w ··· O_w)]. The Gaussian height was initially set at 1 kcal/mol, lowering it to 0.5 kcal/mol near the crossing (and recrossing) over the TS. The Gaussian with was taken as 0.2 Å for each CV. The Gaussian-like biasing functions were added every 380 MD (45.6 fs) steps. The simulation was stopped once the system recrossed over the transition state. The evolution of the CVs during the metadynamics simulation is provided in Figure S7.

3. References

(1) Ito, T.; Katayama, T.; Hattie, M.; Sakurama, H.; Wada, J.; Suzuki, R.; Ashida, H.; Wakagi, T.; Yamamoto, K.; Stubbs, K. A.; Fushinobu, S. Crystal Structures of a Glycoside Hydrolase Family 20 Lacto-*N*-Biosidase from *Bifidobacterium Bifidum*. *J. Biol. Chem.* **2013**, *288*, 11795–11806.

(2) Søndergaard, C. R.; Olsson, M. H. M.; Rostkowski, M.; Jensen, J. H. Improved Treatment of Ligands and Coupling Effects in Empirical Calculation and Rationalization of p*K*a Values. *J. Chem. Theory Comput.* **2011**, *7*, 2284–2295.

(3) Olsson, M. H. M.; Søndergaard, C. R.; Rostkowski, M.; Jensen, J. H. PROPKA3: Consistent Treatment of Internal and Surface Residues in Empirical p*K*a Predictions. *J. Chem. Theory Comput*. **2011**, 7, 525–537.

(4) Anandakrishnan, R.; Aguilar, B.; Onufriev, A. v. H++ 3.0: Automating p*K* Prediction and the Preparation of Biomolecular Structures for Atomistic Molecular Modeling and Simulations. *Nucleic Acids Res.* **2012**, *40*, 537–541.

(5) Trott, O.; Olson, A. J. AutoDock Vina: Improving the Speed and Accuracy of Docking with a New Scoring Function, Efficient Optimization, and Multithreading. *J. Comput. Chem.* **2010**, *31*, 455–461.

(6) Case, D. A.; Ben-Shalom, I. Y.; Brozell, S. R.; Cerutti, D. S.; Cheatham, III, T. E.; Cruzeiro, V. W. D.; Darden, T. A.; Duke, R. E.; Ghoreishi, D.; Gilson, M. K.; Gohlke, H.; Goetz, A. W.; Greene, D.; Harris, R.; Homeyer, N.; Huang, Y.; Izadi, S.; Kovalenko, A.; Kurtzman, T.; Lee, T. S.; LeGrand, S.; Li, P.; Lin, C.; Liu, J.; Luchko, T.; Luo, R.;

Mermelstein, D. J.; Merz, K. M.; Miao, Y.; Monard, G.; Nguyen, C.; Nguyen, H.; Omelyan, I.; Onufriev, A.; Pan, F.; Qi, R.; Roe, D. R.; Roitberg, A.; Sagui, C.; Schott-Verdugo, S.; Shen, J.; Simmerling, C. L.; Smith, J.; Salomon Ferrer, R.; Swails, J.; Walker, R. C.; Wang, J.; Wei, H.; Wolf, R. M.; Wu, X.; Xiao, L.; York, D. M.; Kollman, P. A. AMBER 2018, University of California, San Francisco, **2018**

(7) Hornak, V.; Abel, R.; Okur, A.; Strockbine, B.; Roitberg, A.; Simmerling, C. Comparison of Multiple Amber Force Fields and Development of Improved Protein Backbone Parameters. *Proteins* **2006**, *65*, 712–725.

(8) Kirschner, K. N.; Yongye, A. B.; Tschampel, S. M.; González-Outeiriño, J.; Daniels, C. R.; Foley, B. L.; Woods, R. J. GLYCAM06: A Generalizable Biomolecular Force Field. Carbohydrates. *J. Comput. Chem.* **2008**, *29*, 622–655.

(9) Jorgensen, W. L.; Chandrasekhar, J.; Madura, J. D.; Impey, R. W.; Klein, M. L. Comparison of Simple Potential Functions for Simulating Liquid Water. *J. Chem. Phys.* **1998**, *79*, 926–935.

(10) CPMD program, Copyright IBM Corp. 1990-2003, Copyright MPI für Festkörperforschung, Stuttgart 1997-2001. URL: http://www.cpmd.org (accessed 01/10/2021).

(11) Raich, L.; Nin-Hill, A.; Ardèvol, A.; Rovira, C. Enzymatic Cleavage of Glycosidic Bonds: Strategies on How to Set Up and Control a QM/MM Metadynamics Simulation. *Methods Enzymol.* **2016**, *577*, 159–183.

(12) Laio, A.; VandeVondele, J.; Rothlisberger, U. A Hamiltonian Electrostatic Coupling Scheme for Hybrid Car–Parrinello Molecular Dynamics Simulations. *J. Chem. Phys.* **2002**, *116*, 6941–6947.

(13) Car, R.; Parrinello, M. Unified Approach for Molecular Dynamics and Density-Functional Theory. *Phys. Rev. Lett.* **1985**, *55*, 2471–2474.

(14) Perdew, J. P.; Burke, K.; Ernzerhof, M. Generalized Gradient Approximation Made Simple. *Phys. Rev. Lett.* **1996**, 77, 3865–3868.

(15) Marianski, M.; Supady, A.; Ingram, T.; Schneider, M.; Baldauf, C. Assessing the Accuracy of Across-the-Scale Methods for Predicting Carbohydrate Conformational Energies for the Examples of Glucose and α-Maltose. *J. Chem. Theory Comput.* **2016**, *12*, 6157–6168.

(16) Ardèvol, A.; Rovira, C. Reaction Mechanisms in Carbohydrate-Active Enzymes: Glycoside Hydrolases and Glycosyltransferases. Insights from *ab Initio* Quantum Mechanics/Molecular Mechanics Dynamic Simulations. *J. Am. Chem. Soc*. **2015**, *137*, 7528–7547.

(17) Teze, D.; Coines, J.; Raich, L.; Kalichuk, V.; Solleux, C.; Tellier, C.; André-Miral, C.; Svensson, B.; Rovira, C. A Single Point Mutation Converts GH84 *O*-GlcNAc Hydrolases into Phosphorylases: Experimental and Theoretical Evidence. *J. Am. Chem. Soc.* **2020**, *142*, 2120–2124.

(18) Nin-Hill, A.; Rovira, C. The Catalytic Reaction Mechanism of the *β*-Galactocerebrosidase Enzyme Deficient in Krabbe Disease. *ACS Catal.* **2020**, *10*, 12091–12097.

(19) B. Morais, M. A.; Coines, J.; Domingues, M. N.; S Pirolla, R. A.; C Tonoli, C. C.; Santos, C. R.; L Correa, J. B.; Gozzo, F. C.; Rovira, C.; Murakami, M. T. Two Distinct Catalytic Pathways for GH43 Xylanolytic Enzymes Unveiled by X-Ray and QM/MM Simulations. *Nat. Commun.* **2021**, 12, 367.

(20) Troullier, N.; Martins, J. L. Efficient Pseudopotentials for Plane-Wave Calculations. *Phys. Rev. B.* **1991**, 43, 1993–2006.

(21) Nosé, S. A Unified Formulation of the Constant Temperature Molecular Dynamics Methods. *J. Chem. Phys.* **1984**, *81*, 511–519.

(22) Bonomi, M.; Bussi, G.; Camilloni, C.; Tribello, G. A.; Banáš, P.; Barducci, A.; Bernetti, M.; Bolhuis, P. G.; Bottaro, S.; Branduardi, D.; Capelli, R.; Carloni, P.; Ceriotti, M.; Cesari, A.; Chen, H.; Chen, W.; Colizzi, F.; De, S.; de La Pierre, M.; Donadio, D.; Drobot, V.; Ensing, B.; Ferguson, A. L.; Filizola, M.; Fraser, J. S.; Fu, H.; Gasparotto, P.; Gervasio, F. L.; Giberti, F.; Gil-Ley, A.; Giorgino, T.; Heller, G. T.; Hocky, G. M.; Iannuzzi, M.; Invernizzi, M.; Jelfs, K. E.; Jussupow, A.; Kirilin, E.; Laio, A.; Limongelli, V.; Lindorff-Larsen, K.; Löhr, T.; Marinelli, F.; Martin-Samos, L.; Masetti, M.; Meyer, R.; Michaelides, A.; Molteni, C.; Morishita, T.; Nava, M.; Paissoni, C.; Papaleo, E.; Parrinello, M.; Pfaendtner, J.; Piaggi, P.; Piccini, G. M.; Pietropaolo, A.; Pietrucci, F.; Pipolo, S.; Provasi, D.; Quigley, D.; Raiteri, P.; Raniolo, S.; Rydzewski, J.; Salvalaglio, M.; Sosso, G. C.; Spiwok, V.; Šponer, J.; Swenson, D. W. H.; Tiwary, P.; Valsson, O.; Vendruscolo, M.; Voth,

G. A.; White, A. Promoting Transparency and Reproducibility in Enhanced Molecular Simulations. *Nat. Methods*. **2019**, *16*, 670–673.
(23) Tribello, G. A.; Bonomi, M.; Branduardi, D.; Camilloni, C.; Bussi, G. PLUMED 2: New Feathers for an Old Bird. *Comput. Phys. Commun.* **2014**, *185*, 604–613.
(24) Ensing, B.; Laio, A.; Parrinello, M.; Klein, M. L. A Recipe for the Computation of the Free Energy Barrier and the Lowest Free Energy Path of Concerted Reactions. *J. Phys. Chem. B*. **2005**, *109*, 6676–6687.
(25) Humphrey, W.; Dalke, A.; Schulten, K. "VMD - Visual Molecular Dynamics", *J. Molec. Graphics*. **1996**, *14*, 33–38.
(26) Roe, D. R.; Cheatham, T. E. PTRAJ and CPPTRAJ: Software for Processing and Analysis of Molecular Dynamics Trajectory Data. *J. Chem. Theory Comput.* **2013**, *9*, 3084–3095.
(27) Marcos-Alcalde, I.; Lopez-Viñas, E.; Gómez-Puertas, P. MEPSAnd: Minimum Energy Path Surface Analysis over n-dimensional surfaces. *Bioinformatics.* **2020**, 36, 956–958.
(28) Maier, J. A.; Martinez, C.; Kasavajhala, K.; Wickstrom, L.; Hauser, K. E.; Simmerling, C. FF14SB: Improving the Accuracy of Protein Side Chain and Backbone Parameters from FF99SB. *J. Chem. Theory Comput.* **2015**, *11*, 3696–3713.
(29) Gaussian 09, Revision D.01, Frisch, M. J.; Trucks, G. W.; Schlegel, H. B.; Scuseria, G. E.; Robb, M. A.; Cheeseman, J. R.; Scalmani, G.; Barone, V.; Petersson, G. A.; Nakatsuji, H.; Li, X.; Caricato, M.; Marenich, A.; Bloino, J.; Janesko, B. G.; Gomperts, R.; Mennucci, B.; Hratchian, H. P.; Ortiz, J. V.; Izmaylov, A. F.; Sonnenberg, J. L.; Williams-Young, D.; Ding, F.; Lipparini, F.; Egidi, F.; Goings, J.; Peng, B.; Petrone, A.; Henderson, T.; Ranasinghe, D.; Zakrzewski, V. G.; Gao, J.; Rega, N.; Zheng, G.; Liang, W.; Hada, M.; Ehara, M.; Toyota, K.; Fukuda, R.; Hasegawa, J.; Ishida, M.; Nakajima, T.; Honda, Y.; Kitao, O.; Nakai, H.; Vreven, T.; Throssell, K.; Montgomery Jr., J. A.; Peralta, J. E.; Ogliaro, F.; Bearpark, M.; Heyd, J. J.; Brothers, E.; Kudin, K. N.; Staroverov, V. N.; Keith, T.; Kobayashi, R.; Normand, J.; Raghavachari, K.; Rendell, A.; Burant, J. C.; Iyengar, S. S.; Tomasi, J.; Cossi, M.; Millam, J. M.; Klene, M.; Adamo, C.; Cammi, R.; Ochterski, J. W.; Martin, R. L.; Morokuma, K.; Farkas, O.; Foresman, J. B.; Fox, D. J. Gaussian, Inc., Wallingford CT, **2016**.

(30) Wang, J.; Wolf, R. M.; Caldwell, J. W.; Kollman, P. A.; Case, D. A. Development and Testing of a General Amber Force Field. *J. Comput. Chem.* **2004**, *25*, 1157–1174.

(31) Wang, J.; Wang, W.; Kollman, P. A.; Case, D. A. Automatic Atom Type and Bond Type Perception in Molecular Mechanical Calculations. *J. Mol. Graph.* **2006**, *25*, 247–260.

4. Supporting tables

Table S1. Relevant active site distances at the three main states of the first reaction step. Distances are given as averages with standard deviation.

	State		
Distance	**R**	**TS1**	**INT**
C1 - O5	1.43 ± 0.04	1.33 ± 0.04	1.32 ± 0.03
C1 - O_{2N}	2.89 ± 0.12	2.59 ± 0.13	1.85 ± 0.09
C1 - O1	1.47 ± 0.05	1.99 ± 0.15	2.70 ± 0.09
H_{Glu321} - O_{Glu321}	1.01 ± 0.03	1.14 ± 0.04	1.69 ± 0.06
H_{Glu321} - O1	2.33 ± 0.18	1.39 ± 0.05	1.02 ± 0.02
H_{His263} – O_{Asp320}	1.88 ± 0.11	2.04 ± 0.17	3.19 ± 0.67
H_{His263} - O_{Glu321}	2.84 ± 0.23	2.41 ± 0.27	1.93 ± 0.16
N – H	1.04 ± 0.02	1.02 ± 0.03	1.13 ± 0.08
H - O_{Asp320}	1.90 ± 0.12	1.83 ± 0.08	1.45 ± 0.14
H_{Tyr419} – O_{2N}	1.85 ± 0.14	1.76 ± 0.15	1.99 ± 0.18

Table S2. Relevant active site distances at the three main states of the second reaction step. Distances are given as averages with standard deviation.

	State		
distance	**INT**	**TS2**	**P**
C1 - O_w	3.45 ± 0.20	2.30 ± 0.15	1.46 ± 0.04
C1 – O_{2N}	1.70 ± 0.11	2.35 ± 0.16	2.95 ± 0.10
O_{Glu321} - H_w	1.79 ± 0.14	1.53 ± 0.06	1.01 ± 0.03
H_w - O_w	1.01 ± 0.03	1.07 ± 0.03	1.85 ± 0.14
O_{Asp320} – H	1.65 ± 0.11	1.91 ± 0.13	2.14 ± 0.22
H – N	1.07 ± 0.04	1.05 ± 0.03	1.03 ± 0.03
C1 – O5	1.34 ± 0.03	1.29 ± 0.03	1.41 ± 0.03
O_{2N} - H_{Tyr419}	2.05 ± 0.19	1.96 ± 0.14	1.86 ± 0.14
H_{His263} – O_{Asp320}	3.18 ± 0.47	2.15 ± 0.34	2.08 ± 0.24
H_{His263} – O_{Glu321}	2.49 ± 0.84	2.69 ± 0.38	2.66 ± 0.40

5. Supporting figures

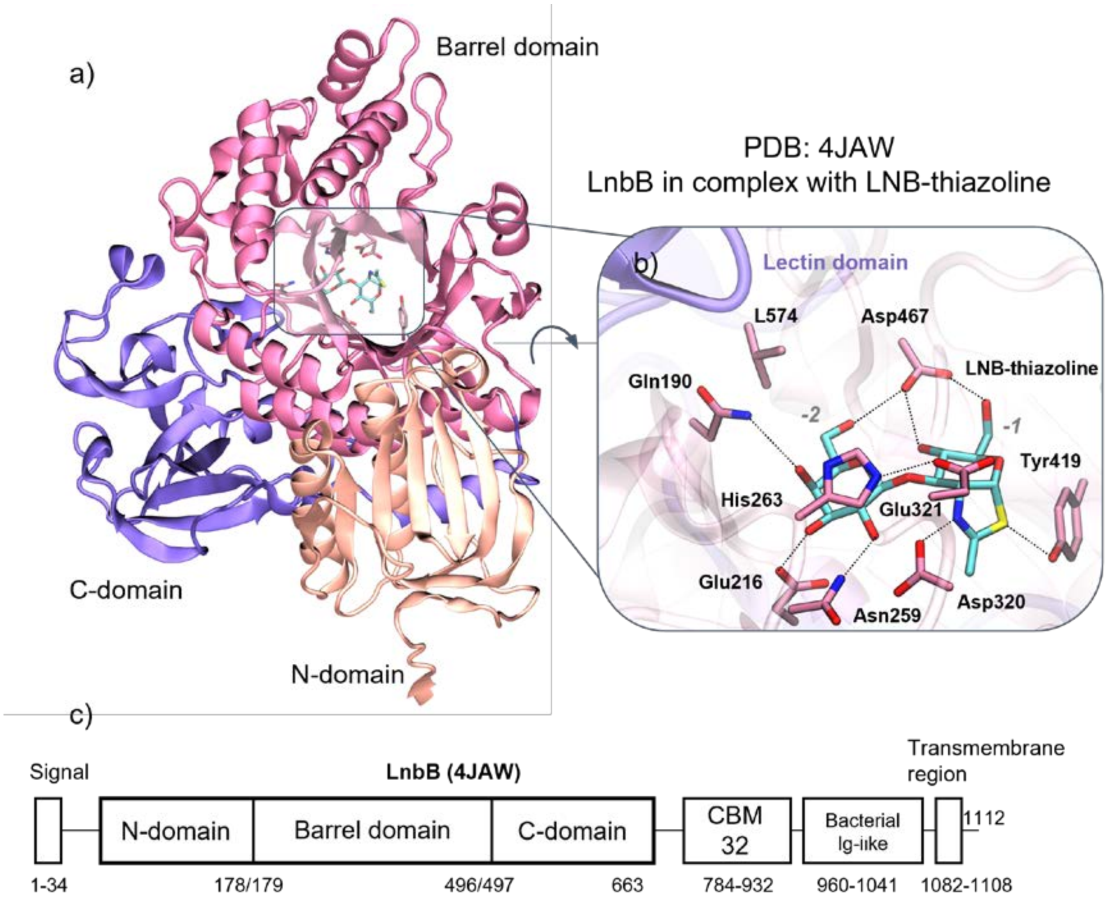

Figure S1. (a) LnbB crystal structure in complex with the inhibitor LNB-thiazoline (PDB accession code 4JAW, resolution 1.80 Å). (b) The active site host two sugar units (at -*2* and -*1* subsites), in contrast with other GH20s. (c) The protein structure includes a N-domain, a barrel domain that contains the active site and a C-domain.

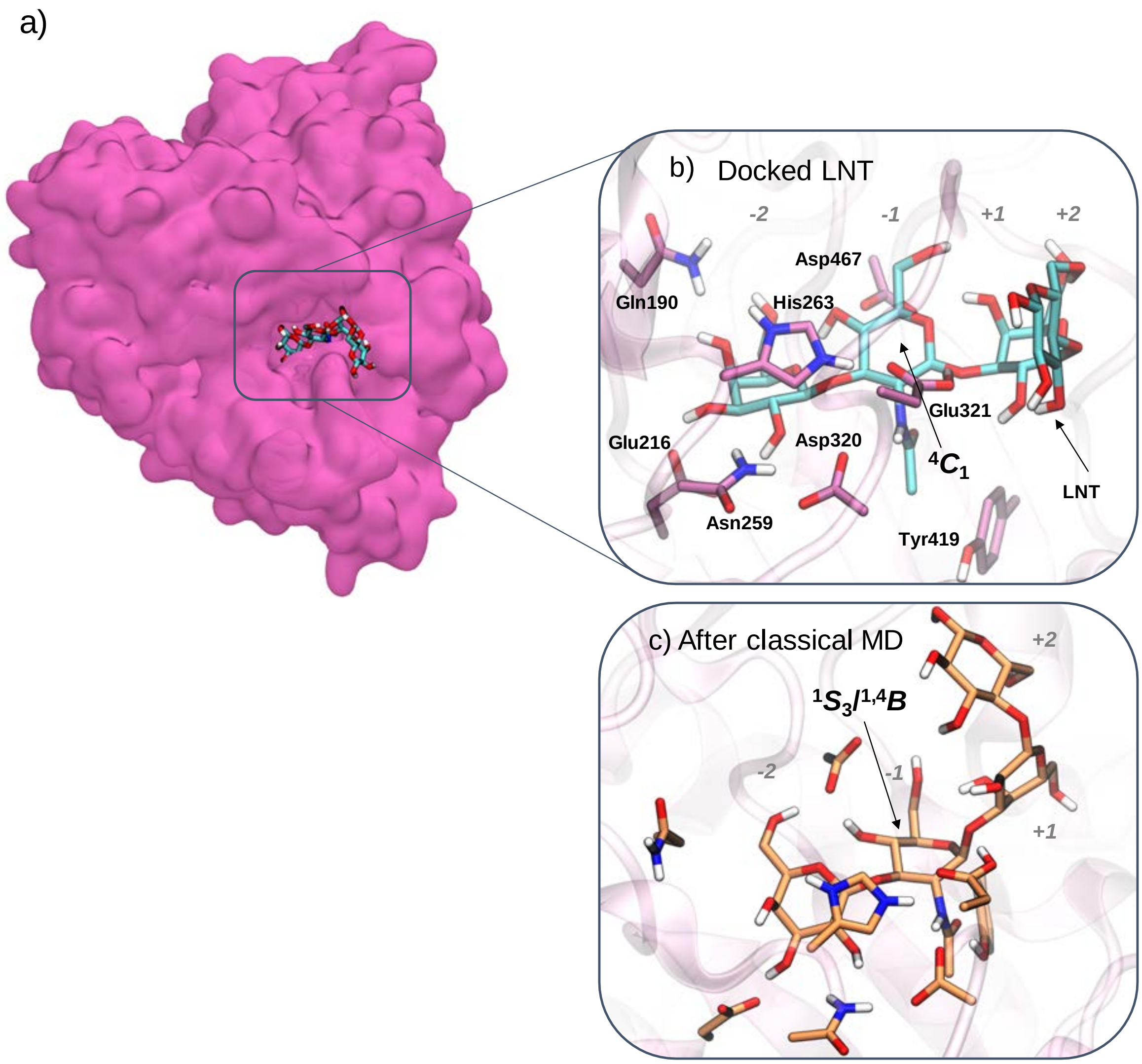

Figure S2. (a) LnbB structure (fom PDB 4JAW) in complex with a lacto-*N*-tetraose (LNT) substrate (blue sticks), obtained by molecular docking. (b,c) Detailed view of the active site showing the residues of the active site involved in the binding of LNT. Comparison of the substrate accommodation at the active site before classical MD (blue sticks) and after MD (orange sticks). The conformation of the sugar unit at the -*1* subsite evolves from $^{4}C_{1}$ to a distorted $^{1}S_{3}$ / $^{1,4}B$ conformation.

a)

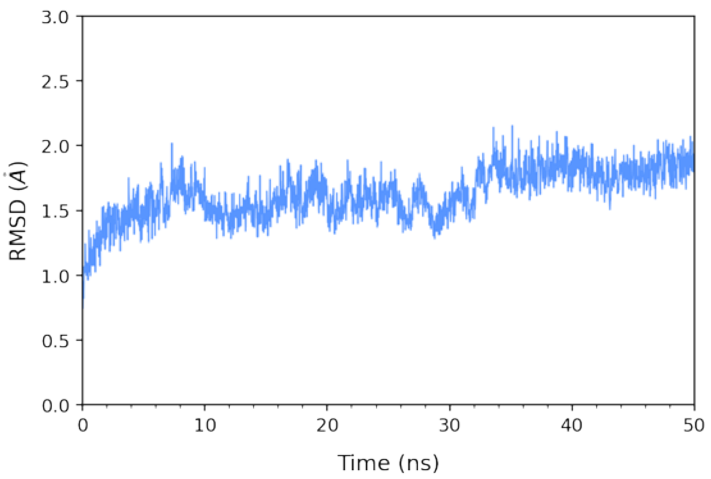

b)

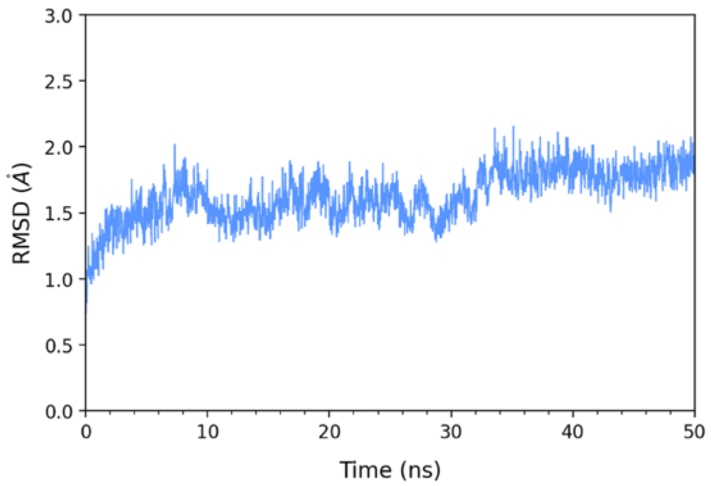

Figure S3. RMSD evolution of the backbone C atoms along classical MD simulation of the (a) Michaelis complex and (b) reaction intermediate.

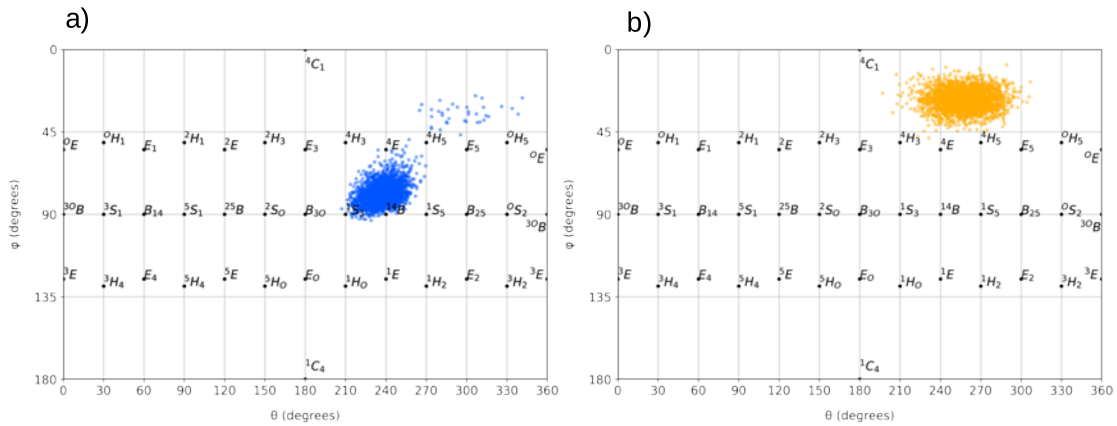

Figure S4. Conformations of the pyranose sugar at the *-1* enzyme subsite sampled during the classical MD simulations. (a) Enzyme-LNT complex, (b) enzyme-LNB reaction intermediate.

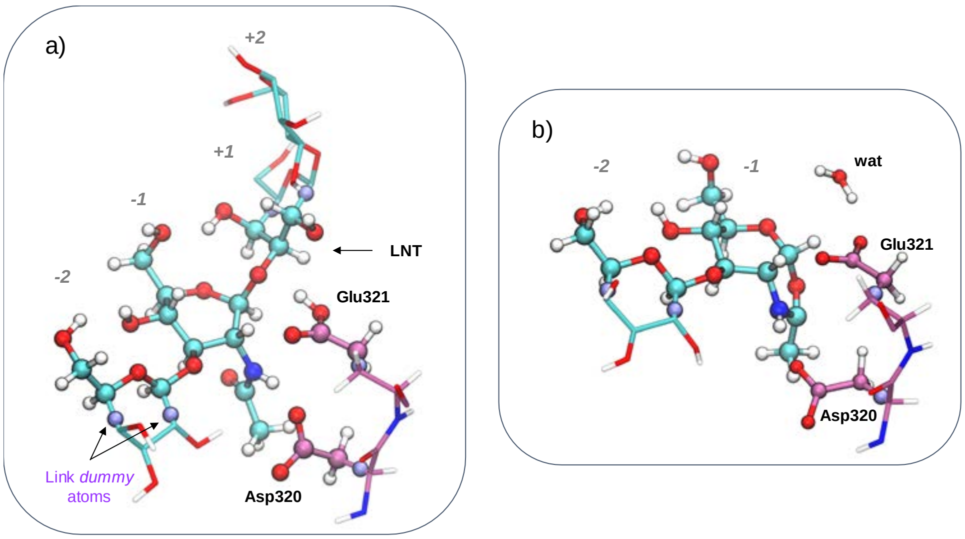

Figure S5. Active site view of the MD structure used to initiate QM/MM MD simulations of (a) the Michaelis complex (67 QM atoms and 111345 MM atoms) and (b) reaction intermediate (56 QM atoms and 111354 MM atoms). The atoms included in the QM calculations are depicted as spheres. Link atoms are represented as violet spheres.

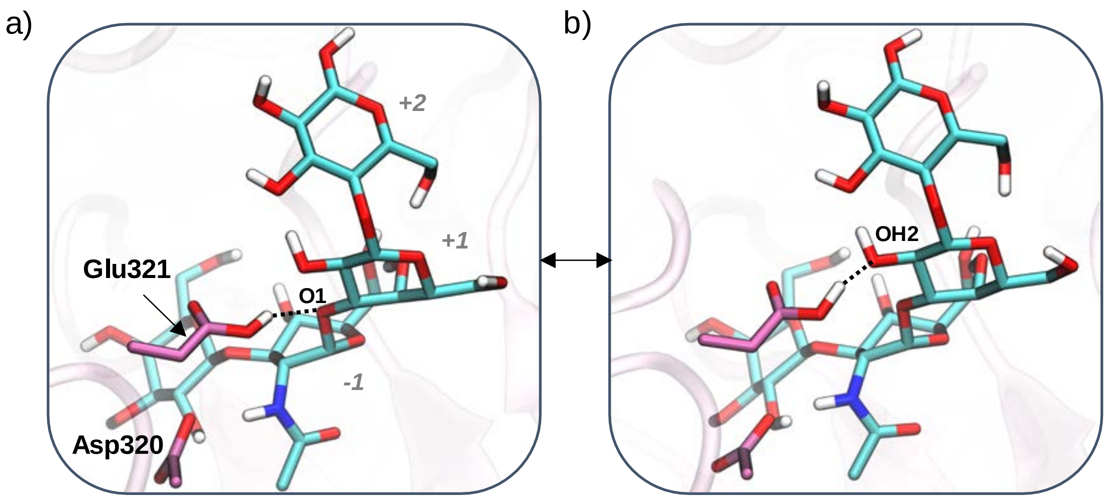

Figure S6. Two alternative orientations of the acid/base residue (Glu321) visited during classical and QM/MM MD simulations of the Michaelis complex. Glu321 is either interacting with the glycosidic oxygen or the 2-hydrolxyl group of the galactose unit at the *+1* subsite.

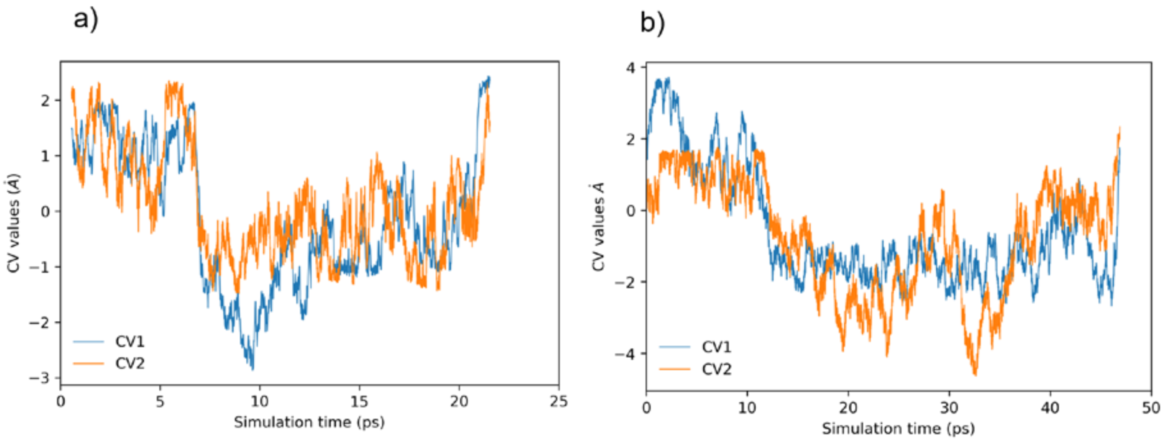

Figure S7. Evolution of the collective variables during the metadynamics reactions of the (a) first reaction step, (b) second reaction step.

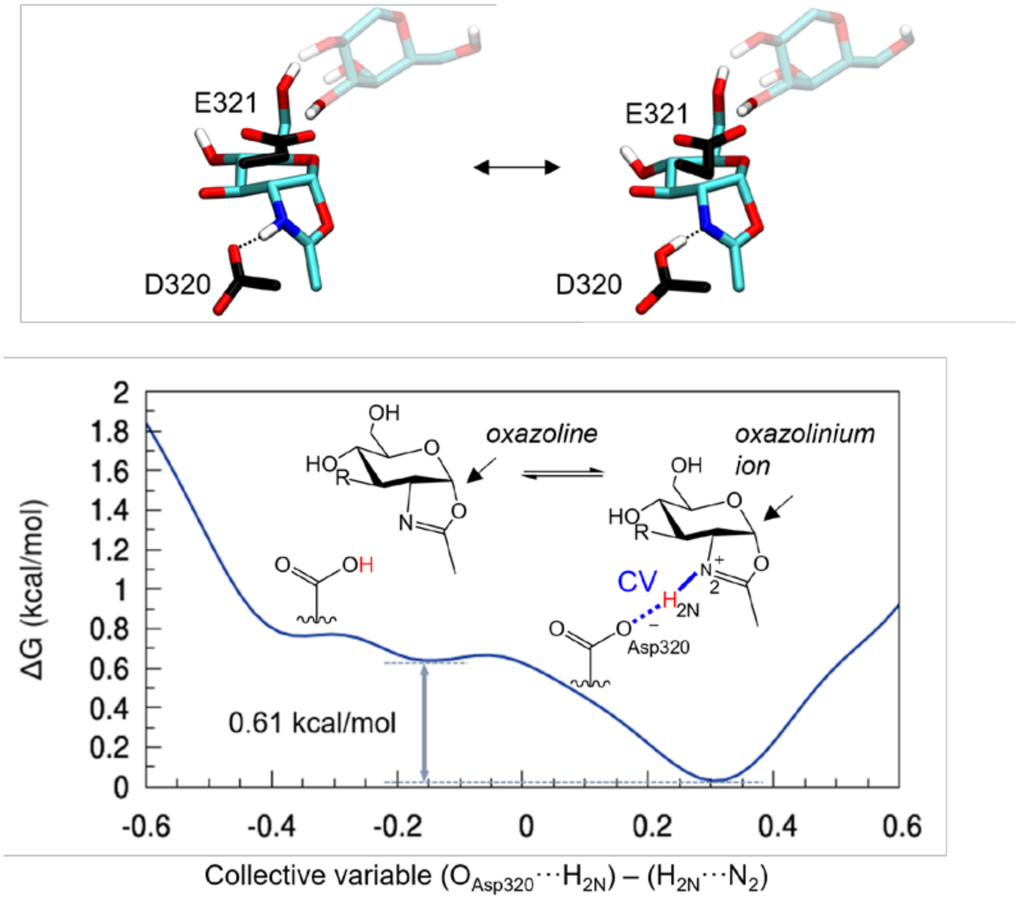

Figure S8. Free energy profile for the conversion between oxazoline and oxazolinium ion form of the reaction intermediate (INT) obtained from QM/MM metadynamics simulations.

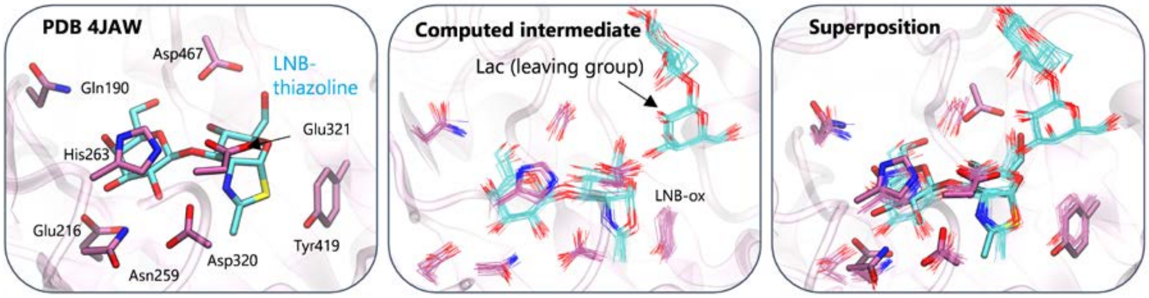

Figure S9. Comparison between the experimental structure of LnbB in complex with LNB-thiazoline (PDB 4JAW) and the computed reaction intermediate after the first reaction step. Computed structures of the intermediate have been extracted from the minimum of the reaction free energy landscape that corresponds to the reaction intermediate (the ensemble of structures represent the dynamical motion of the system at 300K).

4. Cartesian coordinates of the main states along the reaction pathway

The Cartesian coordinates (PDB format) of the atoms included in the QM region in states MC’, MC, TS1, INT and P (representative structures shown in Figure 3C) are listed below.

MC’

```
ATOM      1  CB  ASP X 281       6.072  12.364  14.252  0.00  0.00
ATOM      2  HB2 ASP X 281       6.326  13.351  13.874  0.00  0.00
ATOM      3  HB3 ASP X 281       6.814  12.058  14.940  0.00  0.00
ATOM      4  CG  ASP X 281       5.964  11.245  13.180  0.00  0.00
ATOM      5  OD1 ASP X 281       5.905  10.057  13.633  0.00  0.00
ATOM      6  OD2 ASP X 281       5.982  11.611  11.885  0.00  0.00
ATOM      7  CG  GLH X 282       4.065  13.629  10.487  0.00  0.00
ATOM      8  HG2 GLH X 282       4.076  14.727  10.391  0.00  0.00
ATOM      9  HG3 GLH X 282       4.797  13.205  11.211  0.00  0.00
ATOM     10  CD  GLH X 282       4.569  13.082   9.114  0.00  0.00
ATOM     11  OE1 GLH X 282       4.137  12.219   8.374  0.00  0.00
ATOM     12  OE2 GLH X 282       5.649  13.839   8.781  0.00  0.00
ATOM     13  HE2 GLH X 282       5.777  13.967   7.775  0.00  0.00
ATOM     14  C1  0LB X 624       6.891   7.450   9.076  0.00  0.00
ATOM     15  H1  0LB X 624       7.985   7.031   8.973  0.00  0.00
ATOM     16  O5  0LB X 624       6.184   6.888   7.966  0.00  0.00
ATOM     17  C5  0LB X 624       6.313   5.517   7.748  0.00  0.00
ATOM     18  H5  0LB X 624       7.362   5.200   7.796  0.00  0.00
ATOM     19  C6  0LB X 624       5.702   5.052   6.453  0.00  0.00
ATOM     20  H62 0LB X 624       4.741   5.599   6.244  0.00  0.00
ATOM     21  H61 0LB X 624       5.426   4.047   6.341  0.00  0.00
ATOM     22  O6  0LB X 624       6.605   5.282   5.230  0.00  0.00
ATOM     23  H6O 0LB X 624       6.562   6.252   5.029  0.00  0.00
ATOM     24  C1  3YB X 625       9.037  11.943   7.947  0.00  0.00
ATOM     25  H1  3YB X 625       9.595  12.638   8.608  0.00  0.00
ATOM     26  O5  3YB X 625      10.120  11.246   7.291  0.00  0.00
ATOM     27  C5  3YB X 625       9.855   9.930   6.660  0.00  0.00
ATOM     28  H5  3YB X 625      10.639   9.271   7.130  0.00  0.00
ATOM     29  C6  3YB X 625      10.207  10.117   5.173  0.00  0.00
ATOM     30  H62 3YB X 625      10.419   9.167   4.632  0.00  0.00
ATOM     31  H61 3YB X 625      11.190  10.543   5.208  0.00  0.00
ATOM     32  O6  3YB X 625       9.163  10.955   4.415  0.00  0.00
ATOM     33  H6O 3YB X 625       9.225  10.864   3.420  0.00  0.00
ATOM     34  C4  3YB X 625       8.450   9.345   7.018  0.00  0.00
ATOM     35  H4  3YB X 625       7.698   9.881   6.433  0.00  0.00
ATOM     36  O4  3YB X 625       8.468   7.991   6.612  0.00  0.00
ATOM     37  H4O 3YB X 625       7.706   7.909   5.937  0.00  0.00
ATOM     38  C3  3YB X 625       8.234   9.598   8.520  0.00  0.00
ATOM     39  H3  3YB X 625       9.064   9.048   9.035  0.00  0.00
ATOM     40  C2  3YB X 625       8.093  11.075   8.855  0.00  0.00
ATOM     41  H2  3YB X 625       7.096  11.425   8.651  0.00  0.00
ATOM     42  N2  3YB X 625       8.331  11.379  10.297  0.00  0.00
ATOM     43  H2N 3YB X 625       7.502  11.630  10.835  0.00  0.00
```

```
ATOM     44  C2N 3YB X 625       9.543  11.060  10.786  0.00  0.00
ATOM     45  O2N 3YB X 625      10.448  10.585  10.061  0.00  0.00
ATOM     46  CME 3YB X 625       9.672  11.234  12.286  0.00  0.00
ATOM     47  H3M 3YB X 625       9.399  10.328  12.861  0.00  0.00
ATOM     48  H2M 3YB X 625       9.138  12.152  12.683  0.00  0.00
ATOM     49  H1M 3YB X 625      10.724  11.392  12.572  0.00  0.00
ATOM     50  O3  3YB X 625       6.894   8.881   9.074  0.00  0.00
ATOM     51  C4  3LB X 626       9.933  14.002   6.060  0.00  0.00
ATOM     52  H4  3LB X 626      10.570  13.542   6.817  0.00  0.00
ATOM     53  O4  3LB X 626       9.843  13.355   4.840  0.00  0.00
ATOM     54  H4O 3LB X 626       9.479  12.445   4.971  0.00  0.00
ATOM     55  C3  3LB X 626       8.615  13.979   6.701  0.00  0.00
ATOM     56  H3  3LB X 626       8.636  14.424   7.702  0.00  0.00
ATOM     57  C2  3LB X 626       7.606  14.651   5.847  0.00  0.00
ATOM     58  H2  3LB X 626       7.512  14.023   4.953  0.00  0.00
ATOM     59  O2  3LB X 626       6.248  14.656   6.451  0.00  0.00
ATOM     60  H2O 3LB X 626       5.704  14.908   5.657  0.00  0.00
ATOM     61  O3  3LB X 626       8.093  12.661   7.038  0.00  0.00
```

MC

```
ATOM      1  CB  ASP X 281       6.019  12.461  14.524  0.00  0.00
ATOM      2  HB2 ASP X 281       6.100  13.314  13.891  0.00  0.00
ATOM      3  HB3 ASP X 281       6.827  12.373  15.226  0.00  0.00
ATOM      4  CG  ASP X 281       5.975  11.131  13.551  0.00  0.00
ATOM      5  OD1 ASP X 281       5.771  10.027  14.076  0.00  0.00
ATOM      6  OD2 ASP X 281       6.024  11.416  12.325  0.00  0.00
ATOM      7  CG  GLH X 282       4.273  13.757  10.892  0.00  0.00
ATOM      8  HG2 GLH X 282       4.226  14.882  11.041  0.00  0.00
ATOM      9  HG3 GLH X 282       4.875  13.312  11.746  0.00  0.00
ATOM     10  CD  GLH X 282       5.006  13.187   9.659  0.00  0.00
ATOM     11  OE1 GLH X 282       5.030  12.045   9.265  0.00  0.00
ATOM     12  OE2 GLH X 282       5.850  14.244   9.163  0.00  0.00
ATOM     13  HE2 GLH X 282       6.377  13.845   8.419  0.00  0.00
ATOM     14  C1  0LB X 624       6.967   7.451   8.846  0.00  0.00
ATOM     15  H1  0LB X 624       8.024   7.145   8.818  0.00  0.00
ATOM     16  O5  0LB X 624       6.354   6.886   7.661  0.00  0.00
ATOM     17  C5  0LB X 624       6.351   5.450   7.713  0.00  0.00
ATOM     18  H5  0LB X 624       7.419   5.107   7.693  0.00  0.00
ATOM     19  C6  0LB X 624       5.752   4.911   6.426  0.00  0.00
ATOM     20  H62 0LB X 624       4.725   5.340   6.312  0.00  0.00
ATOM     21  H61 0LB X 624       5.723   3.785   6.488  0.00  0.00
ATOM     22  O6  0LB X 624       6.591   5.291   5.310  0.00  0.00
ATOM     23  H6O 0LB X 624       6.368   6.210   5.091  0.00  0.00
ATOM     24  C1  3YB X 625       8.737  12.082   7.879  0.00  0.00
ATOM     25  H1  3YB X 625       9.125  12.809   8.669  0.00  0.00
ATOM     26  O5  3YB X 625       9.837  11.520   7.277  0.00  0.00
ATOM     27  C5  3YB X 625       9.715  10.206   6.543  0.00  0.00
ATOM     28  H5  3YB X 625      10.469   9.589   7.087  0.00  0.00
ATOM     29  C6  3YB X 625      10.110  10.285   5.086  0.00  0.00
ATOM     30  H62 3YB X 625      10.460   9.283   4.670  0.00  0.00
```

ATOM	31	H61	3YB	X	625	10.917	10.947	5.148	0.00	0.00
ATOM	32	O6	3YB	X	625	9.092	10.915	4.349	0.00	0.00
ATOM	33	H6O	3YB	X	625	9.321	10.783	3.371	0.00	0.00
ATOM	34	C4	3YB	X	625	8.356	9.512	6.740	0.00	0.00
ATOM	35	H4	3YB	X	625	7.548	10.048	6.219	0.00	0.00
ATOM	36	O4	3YB	X	625	8.463	8.147	6.287	0.00	0.00
ATOM	37	H4O	3YB	X	625	7.567	8.054	5.770	0.00	0.00
ATOM	38	C3	3YB	X	625	8.114	9.556	8.240	0.00	0.00
ATOM	39	H3	3YB	X	625	9.035	9.159	8.729	0.00	0.00
ATOM	40	C2	3YB	X	625	7.860	11.032	8.618	0.00	0.00
ATOM	41	H2	3YB	X	625	6.755	11.099	8.413	0.00	0.00
ATOM	42	N2	3YB	X	625	8.048	11.226	10.051	0.00	0.00
ATOM	43	H2N	3YB	X	625	7.260	11.074	10.718	0.00	0.00
ATOM	44	C2N	3YB	X	625	9.222	11.179	10.707	0.00	0.00
ATOM	45	O2N	3YB	X	625	10.350	11.322	10.132	0.00	0.00
ATOM	46	CME	3YB	X	625	9.255	10.883	12.225	0.00	0.00
ATOM	47	H3M	3YB	X	625	9.161	9.723	12.366	0.00	0.00
ATOM	48	H2M	3YB	X	625	8.391	11.292	12.878	0.00	0.00
ATOM	49	H1M	3YB	X	625	10.224	11.292	12.642	0.00	0.00
ATOM	50	O3	3YB	X	625	6.822	8.930	8.720	0.00	0.00
ATOM	51	C4	3LB	X	626	9.866	14.268	6.113	0.00	0.00
ATOM	52	H4	3LB	X	626	10.562	13.807	6.829	0.00	0.00
ATOM	53	O4	3LB	X	626	9.828	13.596	4.791	0.00	0.00
ATOM	54	H4O	3LB	X	626	9.268	12.786	4.927	0.00	0.00
ATOM	55	C3	3LB	X	626	8.488	14.190	6.678	0.00	0.00
ATOM	56	H3	3LB	X	626	8.437	14.682	7.700	0.00	0.00
ATOM	57	C2	3LB	X	626	7.384	14.863	5.752	0.00	0.00
ATOM	58	H2	3LB	X	626	7.361	14.194	4.968	0.00	0.00
ATOM	59	O2	3LB	X	626	6.115	14.994	6.452	0.00	0.00
ATOM	60	H2O	3LB	X	626	5.394	15.072	5.768	0.00	0.00
ATOM	61	O3	3LB	X	626	7.910	12.937	6.958	0.00	0.00

TS1

ATOM	1	CB	ASP	X	281	6.078	12.199	14.367	0.00	0.00
ATOM	2	HB2	ASP	X	281	6.231	13.068	13.830	0.00	0.00
ATOM	3	HB3	ASP	X	281	6.869	12.141	15.141	0.00	0.00
ATOM	4	CG	ASP	X	281	6.004	10.883	13.365	0.00	0.00
ATOM	5	OD1	ASP	X	281	5.829	9.756	13.919	0.00	0.00
ATOM	6	OD2	ASP	X	281	6.144	11.166	12.136	0.00	0.00
ATOM	7	CG	GLH	X	282	4.570	13.712	10.830	0.00	0.00
ATOM	8	HG2	GLH	X	282	4.585	14.768	10.778	0.00	0.00
ATOM	9	HG3	GLH	X	282	5.140	13.233	11.646	0.00	0.00
ATOM	10	CD	GLH	X	282	5.308	13.187	9.553	0.00	0.00
ATOM	11	OE1	GLH	X	282	5.132	12.080	9.053	0.00	0.00
ATOM	12	OE2	GLH	X	282	6.253	13.977	9.163	0.00	0.00
ATOM	13	HE2	GLH	X	282	6.835	13.585	8.269	0.00	0.00
ATOM	14	C1	0LB	X	624	6.870	7.507	8.851	0.00	0.00
ATOM	15	H1	0LB	X	624	7.929	7.126	8.744	0.00	0.00
ATOM	16	O5	0LB	X	624	6.124	6.904	7.741	0.00	0.00
ATOM	17	C5	0LB	X	624	6.325	5.460	7.727	0.00	0.00

```
ATOM     18  H5  0LB X 624       7.466   5.161   7.779  0.00  0.00
ATOM     19  C6  0LB X 624       5.797   4.936   6.401  0.00  0.00
ATOM     20  H62 0LB X 624       4.725   5.133   6.396  0.00  0.00
ATOM     21  H61 0LB X 624       5.842   3.793   6.311  0.00  0.00
ATOM     22  O6  0LB X 624       6.529   5.442   5.267  0.00  0.00
ATOM     23  H6O 0LB X 624       6.292   6.405   5.080  0.00  0.00
ATOM     24  C1  3YB X 625       8.893  11.905   8.116  0.00  0.00
ATOM     25  H1  3YB X 625       9.475  12.715   8.606  0.00  0.00
ATOM     26  O5  3YB X 625       9.650  11.506   7.060  0.00  0.00
ATOM     27  C5  3YB X 625       9.700  10.139   6.595  0.00  0.00
ATOM     28  H5  3YB X 625      10.358   9.567   7.213  0.00  0.00
ATOM     29  C6  3YB X 625      10.322  10.153   5.175  0.00  0.00
ATOM     30  H62 3YB X 625      10.612   9.070   4.867  0.00  0.00
ATOM     31  H61 3YB X 625      11.158  10.838   5.250  0.00  0.00
ATOM     32  O6  3YB X 625       9.275  10.716   4.316  0.00  0.00
ATOM     33  H6O 3YB X 625       9.570  10.616   3.365  0.00  0.00
ATOM     34  C4  3YB X 625       8.374   9.418   6.827  0.00  0.00
ATOM     35  H4  3YB X 625       7.575   9.863   6.246  0.00  0.00
ATOM     36  O4  3YB X 625       8.651   8.020   6.520  0.00  0.00
ATOM     37  H4O 3YB X 625       7.843   7.727   5.929  0.00  0.00
ATOM     38  C3  3YB X 625       8.089   9.436   8.304  0.00  0.00
ATOM     39  H3  3YB X 625       8.923   8.953   8.887  0.00  0.00
ATOM     40  C2  3YB X 625       8.021  10.906   8.860  0.00  0.00
ATOM     41  H2  3YB X 625       6.947  11.211   8.813  0.00  0.00
ATOM     42  N2  3YB X 625       8.335  10.896  10.226  0.00  0.00
ATOM     43  H2N 3YB X 625       7.632  11.002  10.876  0.00  0.00
ATOM     44  C2N 3YB X 625       9.633  11.116  10.609  0.00  0.00
ATOM     45  O2N 3YB X 625      10.510  10.960   9.758  0.00  0.00
ATOM     46  CME 3YB X 625       9.896  11.431  12.035  0.00  0.00
ATOM     47  H3M 3YB X 625      10.335  10.442  12.373  0.00  0.00
ATOM     48  H2M 3YB X 625       9.035  11.703  12.582  0.00  0.00
ATOM     49  H1M 3YB X 625      10.707  12.098  12.024  0.00  0.00
ATOM     50  O3  3YB X 625       6.860   8.881   8.765  0.00  0.00
ATOM     51  C4  3LB X 626       9.691  14.213   6.002  0.00  0.00
ATOM     52  H4  3LB X 626      10.459  13.701   6.573  0.00  0.00
ATOM     53  O4  3LB X 626       9.532  13.392   4.787  0.00  0.00
ATOM     54  H4O 3LB X 626       9.356  12.370   4.906  0.00  0.00
ATOM     55  C3  3LB X 626       8.341  14.385   6.728  0.00  0.00
ATOM     56  H3  3LB X 626       8.536  15.091   7.510  0.00  0.00
ATOM     57  C2  3LB X 626       7.302  14.960   5.744  0.00  0.00
ATOM     58  H2  3LB X 626       7.088  14.167   5.080  0.00  0.00
ATOM     59  O2  3LB X 626       6.049  15.301   6.432  0.00  0.00
ATOM     60  H2O 3LB X 626       5.404  15.482   5.742  0.00  0.00
ATOM     61  O3  3LB X 626       7.710  13.118   7.329  0.00  0.00
```

INT (obtained from the metadynamics simulation of the first reaction step))

```
ATOM      1  CB  ASP X 281       5.983  11.682  14.383  0.00  0.00
ATOM      2  HB2 ASP X 281       6.392  12.500  13.726  0.00  0.00
ATOM      3  HB3 ASP X 281       6.673  11.428  15.225  0.00  0.00
ATOM      4  CG  ASP X 281       5.890  10.293  13.557  0.00  0.00
```

```
ATOM      5  OD1 ASP X 281       5.029   9.458  13.979  0.00  0.00
ATOM      6  OD2 ASP X 281       6.688  10.057  12.606  0.00  0.00
ATOM      7  CG  GLH X 282       4.273  14.033  10.669  0.00  0.00
ATOM      8  HG2 GLH X 282       4.223  15.155  10.628  0.00  0.00
ATOM      9  HG3 GLH X 282       4.972  13.783  11.491  0.00  0.00
ATOM     10  CD  GLH X 282       4.971  13.547   9.403  0.00  0.00
ATOM     11  OE1 GLH X 282       4.811  12.350   8.918  0.00  0.00
ATOM     12  OE2 GLH X 282       5.758  14.409   8.786  0.00  0.00
ATOM     13  HE2 GLH X 282       6.909  13.768   7.805  0.00  0.00
ATOM     14  C1  0LB X 624       6.833   7.702   9.037  0.00  0.00
ATOM     15  H1  0LB X 624       7.894   7.330   9.080  0.00  0.00
ATOM     16  O5  0LB X 624       6.289   7.174   7.836  0.00  0.00
ATOM     17  C5  0LB X 624       6.542   5.777   7.716  0.00  0.00
ATOM     18  H5  0LB X 624       7.624   5.597   7.757  0.00  0.00
ATOM     19  C6  0LB X 624       6.041   5.224   6.344  0.00  0.00
ATOM     20  H62 0LB X 624       4.990   5.716   6.265  0.00  0.00
ATOM     21  H61 0LB X 624       5.750   4.176   6.452  0.00  0.00
ATOM     22  O6  0LB X 624       6.827   5.319   5.130  0.00  0.00
ATOM     23  H6O 0LB X 624       7.297   6.220   5.177  0.00  0.00
ATOM     24  C1  3YB X 625       9.203  11.965   8.722  0.00  0.00
ATOM     25  H1  3YB X 625       9.171  13.042   8.910  0.00  0.00
ATOM     26  O5  3YB X 625       9.817  11.793   7.600  0.00  0.00
ATOM     27  C5  3YB X 625       9.964  10.298   7.244  0.00  0.00
ATOM     28  H5  3YB X 625      10.817   9.897   7.884  0.00  0.00
ATOM     29  C6  3YB X 625      10.400  10.356   5.775  0.00  0.00
ATOM     30  H62 3YB X 625      10.310   9.294   5.439  0.00  0.00
ATOM     31  H61 3YB X 625      11.489  10.633   5.660  0.00  0.00
ATOM     32  O6  3YB X 625       9.479  11.175   5.103  0.00  0.00
ATOM     33  H6O 3YB X 625       9.337  10.963   4.156  0.00  0.00
ATOM     34  C4  3YB X 625       8.597   9.573   7.282  0.00  0.00
ATOM     35  H4  3YB X 625       7.965  10.018   6.523  0.00  0.00
ATOM     36  O4  3YB X 625       8.750   8.242   6.989  0.00  0.00
ATOM     37  H4O 3YB X 625       8.638   8.140   6.011  0.00  0.00
ATOM     38  C3  3YB X 625       8.087   9.640   8.706  0.00  0.00
ATOM     39  H3  3YB X 625       8.895   9.067   9.294  0.00  0.00
ATOM     40  C2  3YB X 625       8.088  11.039   9.332  0.00  0.00
ATOM     41  H2  3YB X 625       7.034  11.468   9.189  0.00  0.00
ATOM     42  N2  3YB X 625       8.303  10.978  10.744  0.00  0.00
ATOM     43  H2N 3YB X 625       7.548  10.751  11.579  0.00  0.00
ATOM     44  C2N 3YB X 625       9.530  11.086  11.067  0.00  0.00
ATOM     45  O2N 3YB X 625      10.311  11.478  10.084  0.00  0.00
ATOM     46  CME 3YB X 625      10.086  10.839  12.412  0.00  0.00
ATOM     47  H3M 3YB X 625       9.453  11.354  13.075  0.00  0.00
ATOM     48  H2M 3YB X 625      11.168  11.164  12.408  0.00  0.00
ATOM     49  H1M 3YB X 625       9.992   9.715  12.578  0.00  0.00
ATOM     50  O3  3YB X 625       6.825   9.035   8.966  0.00  0.00
ATOM     51  C4  3LB X 626       9.526  14.712   6.082  0.00  0.00
ATOM     52  H4  3LB X 626      10.150  14.373   6.957  0.00  0.00
ATOM     53  O4  3LB X 626       9.685  13.783   4.950  0.00  0.00
ATOM     54  H4O 3LB X 626       9.480  12.874   5.091  0.00  0.00
ATOM     55  C3  3LB X 626       8.092  14.866   6.527  0.00  0.00
ATOM     56  H3  3LB X 626       8.029  15.720   7.280  0.00  0.00
ATOM     57  C2  3LB X 626       7.197  15.185   5.273  0.00  0.00
ATOM     58  H2  3LB X 626       7.389  14.349   4.499  0.00  0.00
```

```
ATOM     59  O2  3LB X 626       5.841  15.380   5.667  0.00  0.00
ATOM     60  H2O 3LB X 626       5.463  14.627   6.193  0.00  0.00
ATOM     61  O3  3LB X 626       7.687  13.616   7.137  0.00  0.00
```

INT (obtained from the crystal structure PDB 4JAW)

```
ATOM      1  CB  ASP X 281      63.753  64.911  59.195  0.00  0.00
ATOM      2  HB2 ASP X 281      63.353  64.994  60.194  0.00  0.00
ATOM      3  HB3 ASP X 281      63.128  65.451  58.420  0.00  0.00
ATOM      4  CG  ASP X 281      63.670  63.424  58.897  0.00  0.00
ATOM      5  OD1 ASP X 281      63.224  62.694  59.856  0.00  0.00
ATOM      6  OD2 ASP X 281      63.999  62.998  57.705  0.00  0.00
ATOM      7  CG  GLU X 282      63.977  62.953  63.118  0.00  0.00
ATOM      8  HG2 GLU X 282      63.737  63.862  63.717  0.00  0.00
ATOM      9  HG3 GLU X 282      63.642  63.070  62.062  0.00  0.00
ATOM     10  CD  GLU X 282      63.180  61.813  63.774  0.00  0.00
ATOM     11  OE1 GLU X 282      63.377  60.613  63.402  0.00  0.00
ATOM     12  OE2 GLU X 282      62.313  62.062  64.616  0.00  0.00
ATOM     13  C1  0LB X 624      62.069  57.393  59.370  0.00  0.00
ATOM     14  H1  0LB X 624      61.165  57.284  58.689  0.00  0.00
ATOM     15  O5  0LB X 624      62.201  56.088  60.083  0.00  0.00
ATOM     16  C5  0LB X 624      62.249  55.022  59.081  0.00  0.00
ATOM     17  H5  0LB X 624      61.430  55.233  58.447  0.00  0.00
ATOM     18  C6  0LB X 624      62.090  53.680  59.686  0.00  0.00
ATOM     19  H62 0LB X 624      62.640  53.587  60.637  0.00  0.00
ATOM     20  H61 0LB X 624      62.351  52.807  59.112  0.00  0.00
ATOM     21  O6  0LB X 624      60.709  53.336  59.929  0.00  0.00
ATOM     22  H6O 0LB X 624      60.254  53.950  60.521  0.00  0.00
ATOM     23  C7  OXI X 625      59.764  62.869  58.748  0.00  0.00
ATOM     24  H4  OXI X 625      60.723  62.884  58.275  0.00  0.00
ATOM     25  H5  OXI X 625      59.362  63.925  58.850  0.00  0.00
ATOM     26  H6  OXI X 625      59.130  62.420  57.952  0.00  0.00
ATOM     27  C6  OXI X 625      59.862  61.994  59.906  0.00  0.00
ATOM     28  O   OXI X 625      58.787  61.656  60.651  0.00  0.00
ATOM     29  N   OXI X 625      61.014  61.371  60.229  0.00  0.00
ATOM     30  H15 OXI X 625      61.934  61.601  59.867  0.00  0.00
ATOM     31  C1  OXI X 625      60.667  60.324  61.245  0.00  0.00
ATOM     32  H7  OXI X 625      61.381  60.192  62.092  0.00  0.00
ATOM     33  C   OXI X 625      59.352  60.873  61.849  0.00  0.00
ATOM     34  H3  OXI X 625      59.569  61.589  62.711  0.00  0.00
ATOM     35  O3  OXI X 625      58.498  59.985  62.347  0.00  0.00
ATOM     36  C4  OXI X 625      58.253  58.784  61.486  0.00  0.00
ATOM     37  C5  OXI X 625      57.099  57.874  62.177  0.00  0.00
ATOM     38  O4  OXI X 625      57.483  57.295  63.413  0.00  0.00
ATOM     39  H12 OXI X 625      57.494  56.331  63.369  0.00  0.00
ATOM     40  H13 OXI X 625      56.163  58.513  62.295  0.00  0.00
ATOM     41  H14 OXI X 625      56.858  56.976  61.519  0.00  0.00
ATOM     42  H10 OXI X 625      57.896  59.141  60.532  0.00  0.00
ATOM     43  C3  OXI X 625      59.573  58.056  61.328  0.00  0.00
ATOM     44  O2  OXI X 625      59.435  56.849  60.609  0.00  0.00
ATOM     45  H11 OXI X 625      59.311  55.986  61.129  0.00  0.00
```

```
ATOM     46  H9  OXI X 625      59.977  57.920  62.345  0.00  0.00
ATOM     47  C2  OXI X 625      60.531  59.058  60.476  0.00  0.00
ATOM     48  H8  OXI X 625      59.939  59.128  59.495  0.00  0.00
ATOM     49  O1  OXI X 625      61.883  58.524  60.250  0.00  0.00
ATOM     50  O   WAT X4617      60.768  59.906  65.614  0.00  0.00
ATOM     51  H1  WAT X4617      61.328  60.656  65.334  0.00  0.00
ATOM     52  H2  WAT X4617      61.090  59.222  64.969  0.00  0.00
ATOM     53  O   WAT X4617      11.476  28.265  23.536  0.00  0.00
ATOM     54  H1  WAT X4617      11.670  27.755  24.299  0.00  0.00
ATOM     55  H2  WAT X4617      12.046  27.884  22.847  0.00  0.00
```

TS2

```
ATOM      1  CB  ASP X 281      64.259  64.887  58.950  0.00  0.00
ATOM      2  HB2 ASP X 281      63.664  65.444  59.672  0.00  0.00
ATOM      3  HB3 ASP X 281      63.867  65.065  57.938  0.00  0.00
ATOM      4  CG  ASP X 281      64.206  63.325  59.089  0.00  0.00
ATOM      5  OD1 ASP X 281      63.621  62.902  60.123  0.00  0.00
ATOM      6  OD2 ASP X 281      64.628  62.541  58.166  0.00  0.00
ATOM      7  CG  GLU X 282      64.050  63.565  63.247  0.00  0.00
ATOM      8  HG2 GLU X 282      64.010  64.075  64.266  0.00  0.00
ATOM      9  HG3 GLU X 282      63.664  64.186  62.391  0.00  0.00
ATOM     10  CD  GLU X 282      63.127  62.325  63.413  0.00  0.00
ATOM     11  OE1 GLU X 282      63.258  61.295  62.747  0.00  0.00
ATOM     12  OE2 GLU X 282      62.155  62.459  64.290  0.00  0.00
ATOM     13  C1  0LB X 624      62.076  57.393  59.614  0.00  0.00
ATOM     14  H1  0LB X 624      61.118  57.402  59.053  0.00  0.00
ATOM     15  O5  0LB X 624      62.288  56.195  60.387  0.00  0.00
ATOM     16  C5  0LB X 624      62.306  54.968  59.549  0.00  0.00
ATOM     17  H5  0LB X 624      61.366  54.905  58.998  0.00  0.00
ATOM     18  C6  0LB X 624      62.322  53.717  60.415  0.00  0.00
ATOM     19  H62 0LB X 624      62.984  53.920  61.254  0.00  0.00
ATOM     20  H61 0LB X 624      62.811  52.845  59.955  0.00  0.00
ATOM     21  O6  0LB X 624      61.005  53.338  60.783  0.00  0.00
ATOM     22  H6O 0LB X 624      60.654  53.973  61.485  0.00  0.00
ATOM     23  C7  OXI X 625      60.093  63.190  58.630  0.00  0.00
ATOM     24  H4  OXI X 625      61.025  63.750  58.856  0.00  0.00
ATOM     25  H5  OXI X 625      59.338  63.955  58.356  0.00  0.00
ATOM     26  H6  OXI X 625      60.252  62.644  57.729  0.00  0.00
ATOM     27  C6  OXI X 625      59.778  62.133  59.698  0.00  0.00
ATOM     28  O   OXI X 625      58.685  61.739  60.181  0.00  0.00
ATOM     29  N   OXI X 625      60.829  61.447  60.115  0.00  0.00
ATOM     30  H15 OXI X 625      61.749  61.897  59.915  0.00  0.00
ATOM     31  C1  OXI X 625      60.639  60.401  61.146  0.00  0.00
ATOM     32  H7  OXI X 625      61.566  60.544  61.720  0.00  0.00
ATOM     33  C   OXI X 625      59.474  60.867  62.057  0.00  0.00
ATOM     34  H3  OXI X 625      59.502  61.903  62.369  0.00  0.00
ATOM     35  O3  OXI X 625      58.515  60.119  62.410  0.00  0.00
ATOM     36  C4  OXI X 625      58.272  58.853  61.640  0.00  0.00
ATOM     37  C5  OXI X 625      57.088  58.073  62.230  0.00  0.00
ATOM     38  O4  OXI X 625      57.392  57.584  63.533  0.00  0.00
ATOM     39  H12 OXI X 625      57.490  56.550  63.437  0.00  0.00
ATOM     40  H13 OXI X 625      56.206  58.691  62.334  0.00  0.00
```

```
ATOM     41  H14 OXI X 625      56.886  57.242  61.567  0.00  0.00
ATOM     42  H10 OXI X 625      58.041  59.275  60.657  0.00  0.00
ATOM     43  C3  OXI X 625      59.597  58.080  61.504  0.00  0.00
ATOM     44  O2  OXI X 625      59.433  56.829  60.894  0.00  0.00
ATOM     45  H11 OXI X 625      59.474  56.142  61.668  0.00  0.00
ATOM     46  H9  OXI X 625      59.809  57.961  62.575  0.00  0.00
ATOM     47  C2  OXI X 625      60.580  58.916  60.675  0.00  0.00
ATOM     48  H8  OXI X 625      60.153  58.889  59.658  0.00  0.00
ATOM     49  O1  OXI X 625      62.003  58.440  60.636  0.00  0.00
ATOM     50  O   WAT X4617      60.630  60.350  63.918  0.00  0.00
ATOM     51  H1  WAT X4617      61.325  61.128  64.187  0.00  0.00
ATOM     52  H2  WAT X4617      60.011  60.231  64.693  0.00  0.00
ATOM     53  O   WAT X4617      10.400  27.323  23.735  0.00  0.00
ATOM     54  H1  WAT X4617      10.807  27.951  24.353  0.00  0.00
ATOM     55  H2  WAT X4617      11.238  27.038  23.242  0.00  0.00
```

P

```
ATOM      1  CB  ASP X 281      63.850  64.525  59.803  0.00  0.00
ATOM      2  HB2 ASP X 281      63.414  64.676  60.830  0.00  0.00
ATOM      3  HB3 ASP X 281      63.028  64.835  59.067  0.00  0.00
ATOM      4  CG  ASP X 281      64.203  63.070  59.669  0.00  0.00
ATOM      5  OD1 ASP X 281      63.720  62.311  60.635  0.00  0.00
ATOM      6  OD2 ASP X 281      64.885  62.619  58.709  0.00  0.00
ATOM      7  CG  GLU X 282      64.409  63.418  63.797  0.00  0.00
ATOM      8  HG2 GLU X 282      64.489  64.091  64.687  0.00  0.00
ATOM      9  HG3 GLU X 282      63.906  64.095  63.006  0.00  0.00
ATOM     10  CD  GLU X 282      63.478  62.238  64.060  0.00  0.00
ATOM     11  OE1 GLU X 282      63.703  61.024  63.928  0.00  0.00
ATOM     12  OE2 GLU X 282      62.212  62.665  64.331  0.00  0.00
ATOM     13  C1  0LB X 624      61.999  57.465  59.684  0.00  0.00
ATOM     14  H1  0LB X 624      61.156  57.409  58.995  0.00  0.00
ATOM     15  O5  0LB X 624      62.118  56.030  60.180  0.00  0.00
ATOM     16  C5  0LB X 624      62.115  54.946  59.146  0.00  0.00
ATOM     17  H5  0LB X 624      61.172  55.160  58.595  0.00  0.00
ATOM     18  C6  0LB X 624      61.994  53.583  59.738  0.00  0.00
ATOM     19  H62 0LB X 624      62.817  53.367  60.482  0.00  0.00
ATOM     20  H61 0LB X 624      62.100  52.840  58.946  0.00  0.00
ATOM     21  O6  0LB X 624      60.713  53.347  60.403  0.00  0.00
ATOM     22  H6O 0LB X 624      60.619  54.103  61.075  0.00  0.00
ATOM     23  C7  OXI X 625      60.025  62.895  58.890  0.00  0.00
ATOM     24  H4  OXI X 625      60.152  62.686  57.831  0.00  0.00
ATOM     25  H5  OXI X 625      60.832  63.536  59.225  0.00  0.00
ATOM     26  H6  OXI X 625      59.145  63.533  58.904  0.00  0.00
ATOM     27  C6  OXI X 625      59.821  61.679  59.845  0.00  0.00
ATOM     28  O   OXI X 625      58.693  61.214  60.003  0.00  0.00
ATOM     29  N   OXI X 625      60.942  61.125  60.493  0.00  0.00
ATOM     30  H15 OXI X 625      61.780  61.691  60.584  0.00  0.00
ATOM     31  C1  OXI X 625      60.686  60.159  61.589  0.00  0.00
ATOM     32  H7  OXI X 625      61.588  60.158  62.183  0.00  0.00
ATOM     33  C   OXI X 625      59.625  60.619  62.609  0.00  0.00
ATOM     34  H3  OXI X 625      59.235  61.587  62.315  0.00  0.00
ATOM     35  O3  OXI X 625      58.476  59.730  62.835  0.00  0.00
```

```
ATOM     36  C4  OXI X 625      58.286  58.763  61.846  0.00  0.00
ATOM     37  C5  OXI X 625      57.127  57.909  62.410  0.00  0.00
ATOM     38  O4  OXI X 625      57.339  57.265  63.727  0.00  0.00
ATOM     39  H12 OXI X 625      57.532  56.267  63.511  0.00  0.00
ATOM     40  H13 OXI X 625      56.288  58.587  62.405  0.00  0.00
ATOM     41  H14 OXI X 625      56.759  57.118  61.725  0.00  0.00
ATOM     42  H10 OXI X 625      57.993  59.289  60.929  0.00  0.00
ATOM     43  C3  OXI X 625      59.467  57.889  61.702  0.00  0.00
ATOM     44  O2  OXI X 625      59.100  56.845  60.771  0.00  0.00
ATOM     45  H11 OXI X 625      58.922  56.071  61.344  0.00  0.00
ATOM     46  H9  OXI X 625      59.812  57.555  62.696  0.00  0.00
ATOM     47  C2  OXI X 625      60.470  58.738  60.970  0.00  0.00
ATOM     48  H8  OXI X 625      59.941  58.832  59.951  0.00  0.00
ATOM     49  O1  OXI X 625      61.803  58.245  60.818  0.00  0.00
ATOM     50  O   WAT X4617      60.053  60.795  64.002  0.00  0.00
ATOM     51  H1  WAT X4617      61.658  61.826  64.208  0.00  0.00
ATOM     52  H2  WAT X4617      60.253  59.887  64.370  0.00  0.00
ATOM     53  O   WAT X4617      10.642  28.115  19.905  0.00  0.00
ATOM     54  H1  WAT X4617      11.343  27.457  19.684  0.00  0.00
ATOM     55  H2  WAT X4617      10.612  28.690  19.175  0.00  0.00
```

P’

```
ATOM      1  CB  ASP X 281      64.051  64.768  59.681  0.00  0.00
ATOM      2  HB2 ASP X 281      63.460  65.233  60.477  0.00  0.00
ATOM      3  HB3 ASP X 281      63.632  65.115  58.687  0.00  0.00
ATOM      4  CG  ASP X 281      63.856  63.260  59.669  0.00  0.00
ATOM      5  OD1 ASP X 281      62.999  62.718  60.478  0.00  0.00
ATOM      6  OD2 ASP X 281      64.639  62.591  58.955  0.00  0.00
ATOM      7  CG  GLU X 282      64.320  63.412  63.508  0.00  0.00
ATOM      8  HG2 GLU X 282      64.268  64.284  64.111  0.00  0.00
ATOM      9  HG3 GLU X 282      63.969  63.640  62.535  0.00  0.00
ATOM     10  CD  GLU X 282      63.445  62.315  64.023  0.00  0.00
ATOM     11  OE1 GLU X 282      63.322  61.142  63.708  0.00  0.00
ATOM     12  OE2 GLU X 282      62.641  62.864  65.004  0.00  0.00
ATOM     13  C1  0LB X 624      62.062  57.174  59.732  0.00  0.00
ATOM     14  H1  0LB X 624      61.105  57.103  59.115  0.00  0.00
ATOM     15  O5  0LB X 624      62.137  55.860  60.422  0.00  0.00
ATOM     16  C5  0LB X 624      62.159  54.802  59.368  0.00  0.00
ATOM     17  H5  0LB X 624      61.206  54.839  58.781  0.00  0.00
ATOM     18  C6  0LB X 624      62.113  53.428  60.003  0.00  0.00
ATOM     19  H62 0LB X 624      62.910  53.390  60.793  0.00  0.00
ATOM     20  H61 0LB X 624      62.281  52.569  59.315  0.00  0.00
ATOM     21  O6  0LB X 624      60.809  53.109  60.531  0.00  0.00
ATOM     22  H6O 0LB X 624      60.455  53.852  61.053  0.00  0.00
ATOM     23  C7  OXI X 625      59.938  62.869  58.834  0.00  0.00
ATOM     24  H4  OXI X 625      59.108  63.582  58.720  0.00  0.00
ATOM     25  H5  OXI X 625      60.245  62.416  57.896  0.00  0.00
ATOM     26  H6  OXI X 625      60.793  63.317  59.233  0.00  0.00
```

ATOM	27	C6	OXI	X	625	59.544	61.738	59.742	0.00	0.00
ATOM	28	O	OXI	X	625	58.379	61.345	59.973	0.00	0.00
ATOM	29	N	OXI	X	625	60.642	61.215	60.313	0.00	0.00
ATOM	30	H15	OXI	X	625	61.520	61.711	60.233	0.00	0.00
ATOM	31	C1	OXI	X	625	60.639	60.156	61.355	0.00	0.00
ATOM	32	H7	OXI	X	625	61.639	60.279	61.862	0.00	0.00
ATOM	33	C	OXI	X	625	59.536	60.397	62.490	0.00	0.00
ATOM	34	H3	OXI	X	625	59.392	61.472	62.523	0.00	0.00
ATOM	35	O3	OXI	X	625	58.276	59.755	62.339	0.00	0.00
ATOM	36	C4	OXI	X	625	58.084	58.637	61.492	0.00	0.00
ATOM	37	C5	OXI	X	625	56.813	57.994	61.874	0.00	0.00
ATOM	38	O4	OXI	X	625	56.874	57.315	63.158	0.00	0.00
ATOM	39	H12	OXI	X	625	56.961	56.232	63.120	0.00	0.00
ATOM	40	H13	OXI	X	625	56.056	58.775	61.987	0.00	0.00
ATOM	41	H14	OXI	X	625	56.580	57.226	61.122	0.00	0.00
ATOM	42	H10	OXI	X	625	57.857	58.932	60.406	0.00	0.00
ATOM	43	C3	OXI	X	625	59.396	57.771	61.321	0.00	0.00
ATOM	44	O2	OXI	X	625	59.165	56.586	60.592	0.00	0.00
ATOM	45	H11	OXI	X	625	59.179	55.741	61.134	0.00	0.00
ATOM	46	H9	OXI	X	625	59.697	57.479	62.321	0.00	0.00
ATOM	47	C2	OXI	X	625	60.523	58.672	60.716	0.00	0.00
ATOM	48	H8	OXI	X	625	60.262	58.828	59.650	0.00	0.00
ATOM	49	O1	OXI	X	625	61.873	58.139	60.733	0.00	0.00
ATOM	50	O	WAT	X4617		60.119	59.914	63.742	0.00	0.00
ATOM	51	H1	WAT	X4617		61.955	62.080	65.303	0.00	0.00
ATOM	52	H2	WAT	X4617		59.465	59.292	64.211	0.00	0.00
ATOM	53	O	WAT	X4617		8.978	27.113	22.556	0.00	0.00
ATOM	54	H1	WAT	X4617		9.315	26.232	22.605	0.00	0.00
ATOM	55	H2	WAT	X4617		8.040	26.932	22.940	0.00	0.00
